# Variability in intestinal drug metabolizing enzymes and transporters in Crohn's disease and potential impact on oral drug absorption

**DOI:** 10.1002/bcp.70019

**Published:** 2025-03-04

**Authors:** Sarah Alrubia, Brahim Achour, Zubida M. Al‐Majdoub, Amin Rostami‐Hodjegan, Jill Barber

**Affiliations:** ^1^ Centre for Applied Pharmacokinetic Research, Division of Pharmacy and Optometry, School of Health Sciences University of Manchester Manchester UK; ^2^ Pharmaceutical Chemistry Department, College of Pharmacy King Saud University Riyadh Saudi Arabia; ^3^ Department of Biomedical and Pharmaceutical Sciences, College of Pharmacy the University of Rhode Island Kingston Rhode Island USA; ^4^ Certara Predictive Technologies, Certara Inc. Sheffield UK

**Keywords:** Crohn's disease, drug‐metabolizing enzymes, intestine, oral drugs, PBPK, pharmacokinetics, proteomics

## Abstract

**Aims:**

The aim of study was to generate quantitative data on the abundance of drug‐metabolizing enzymes and transporters (DMETs) in inflamed and non‐inflamed Crohn's disease (CD) ileum and colon, for incorporation into physiologically based pharmacokinetic (PBPK) models, enabling prediction of oral drugs' pharmacokinetics (PK) perturbation in CD patients.

**Methods:**

Homogenate fractions were processed from 13 inflamed (six ileum and seven colon) and seven non‐inflamed (two ileum and five colon) CD and 10 healthy (five ileum and five colon) tissues from deceased subjects by calcium chelation elution, and protein abundances determined by liquid chromatography–tandem mass spectrometry (LC–MS/MS)‐based proteomics and compared with healthy values. PBPK simulation was applied to predict the potential effect of altered DMET profiles on the PK of oral drugs.

**Results:**

All investigated proteins showed trends for reduced expression in inflamed and non‐inflamed CD samples relative to healthy individuals. Significant downregulation (*P* < 0.05) was observed for CYP3A4, AOX1, NAT1 and several SULTs in inflamed ileum as well as UGT1A10, NAT1, BCRP and several SULTs in inflamed and non‐inflamed colon. Inter‐individual variability was generally higher in CD, with exceptions, for most targets (up to 146%CV in inflamed ileum and up to 169% in histologically normal colon tissues). Integration of abundance data into a verified PBPK model of CD showed a considerable (≥2‐fold; CD predicted relative to healthy predicted) change in systemic drug exposure for 10 drugs examined.

**Conclusions:**

CD inflammation significantly suppresses the expression of intestinal DMETs, which, together with changes in other system parameters, can alter the fate of drugs taken orally in these patients. Virtual patients within a PBPK framework, informed by the measured DMET ranges in the intestine, may serve as a guide for dose adjustment in the absence of dedicated clinical studies.

**What is already known about this subject**

- There are no guidelines on drug dosage adjustment in Crohn's disease (CD) patients.
- Enzyme and transporter expression alterations and significant changes in oral drug pharmacokinetics have been reported in CD.
- Physiologically based pharmacokinetic (PBPK) models for CD predicted pharmacokinetics of investigated drugs but these cannot be generalized to other drugs.

**What this study adds**

- Measured abundance of enzymes and transporters in inflamed and non‐inflamed Crohn's intestine showed significant reduction from healthy baseline.
- Integration of proteomic data into PBPK models highlighted disease impact on oral drugs' pharmacokinetics.
- These data should facilitate predicting exposure to oral drugs in the CD population, when dedicated clinical studies are lacking.

## 1 INTRODUCTION

Crohn's disease (CD) is an inflammatory disease of the ileum and colon,^1^ which alternates between active phases with severe symptoms and inactive remission with few or no symptoms.^2^ It disproportionately affects urban areas, younger Western populations (15–35 year olds), females and smokers.^1^ Because of the early onset of the disease, patients receive prolonged therapy, including oral pharmacotherapy, both for CD and for comorbidities. Drug treatments are rarely curative, and many patients require hospitalization and surgery.^3^, ^4^ Clinical studies have shown altered pharmacokinetics (PK) of oral drugs in CD patients, as the disease may affect several system variables (e.g., intestine and liver enzymes and transporters, serum albumin, blood flow).^5^ These altered PK profiles of oral drugs in CD means that drug exposure cannot be assumed to mirror that of the general population. Despite this possibility, unlike renal or hepatic impairment, there is no specific guidance for assessing potential dosage adjustment in these patients or for studying such changes for drugs entering the market.^6^ Model‐informed precision dosing (MIPD) can improve clinical outcomes; however, these models should be informed by population‐specific alterations with respect to systems data relevant to drugs handling.^7^ Physiologically based pharmacokinetic (PBPK) models use such input on systems parameters, and have been shown to be capable of predicting drug disposition in disease.^8^ Intestinal inflammation can cause morphological changes to the composition of the epithelial layer, including alterations in the expression of drug‐metabolizing enzymes and transporters (DMETs).^9^, ^10^, ^11^, ^12^

Available publications on DMET expression in CD are generally limited to mRNA quantification and immunohistochemistry, often lacking healthy control groups, and covering only a small number of DMETs.^13^, ^14^, ^15^, ^16^, ^17^ Liquid chromatography–tandem mass spectrometry (LC–MS/MS) proteomics, which offers broad coverage and direct protein quantification,^18^ showed significant abundance changes in several DMETs in inflamed and non‐inflamed ileum and colon.^19^ However, these results were generated in pooled samples, which does not allow estimation of variability and range of virtual CD patients within a PBPK framework.

Literature gaps have compromised most of the available studies applying PBPK modelling to predict CD impact.^5^ The work of Effinger et al. is noteworthy^20^ but is limited to the prediction of a single drug (budesonide) in CD without differentiating between active and inactive CD stages. The model was built based on highest and lowest reported parameter values. The level and performance of intestinal CYP3A4 was based on the measured intestinal extraction ratio of CYP3A4 in CD subjects as a direct reflection of its abundance and the blood flow was not considered. Furthermore, the study did not distinguish between the studies where budesonide bioavailability in the CD population is reported to be significantly different from the control and those where it is not. Generalization of available models is therefore not possible because of a lack of systems‐specific information (e.g. intestine and hepatic DMETs' abundance/activity, vascularity and haemodynamic, and blood proteins) in CD patients required for accurate PK prediction. In this study, we aim to address this gap by determining expression changes in ileum and colon, associated with oral drug disposition, from individual inflamed and histologically normal tissues relative to healthy baseline, considering inter‐individual variability. A second aim was to incorporate DMET data into a PBPK CD model and evaluate potential differences in systemic drug exposure between CD and healthy populations for 12 oral drugs where the absorption section of the PBPK model was defined using mechanistic rather empirical absorption rate constants.

## 2 METHODS

### 2.1 Materials

See Supplementary materials.

### 2.2 Intestine samples and donor demographics

Tissue samples were obtained from CD patients undergoing ileocolonic resection with informed consent. These were: (i) fresh‐frozen human intestine mucosal samples from inflamed tissue (I‐CD) and (ii) histologically normal (HN‐CD) tissue, macroscopically normal regions away from the inflamed bowel regions, from ileum (*n* = 6 and 2, respectively), and from colon (*n* = 7 and 5). Tissues were supplied by Manchester Biomedical Research Centre (BRC) Biobank, Manchester University NHS Foundation Trust, Manchester, UK. Prior ethics approval was granted by NRES Committee Northwest—Haydock (19/NW/0644). The average age at surgery was 40 years (range: 18–68 years). The percentage of female subjects was 54%. Demographic and clinical data are presented in Table S1.

The control group included healthy mucosal samples (ileum, *n* = 5; colon, *n* = 5) obtained from healthy deceased subjects and supplied by Caltag Medsystems Limited (Buckingham, UK). Prior ethics approval was granted by the University Research Ethics Committee (UREC), UK (2019‐8120‐12 392). The average age was 48.5 years (range: 30–70 years). The percentage of female subjects was 40%. The average postmortem interval (PMI) was 4.4 h (range: 1–9 h). Demographic information is presented in Table S2. Lack of differences in protein quantification due to potential systematic errors resulting from unparallel sample collection and storage at the two centres is established, see Supplementary materials.

### 2.3 Homogenate preparation for proteomics

Calcium chelation elution of the mucosa cells and cellular homogenate processing was adapted from Harwood et al.^21^ with minor modifications, as previously published.^19^ Details of the sample preparation method are presented in the Supplementary materials.

### 2.4 Liquid chromatography–tandem mass spectrometry (LC–MS/MS)

Samples were diluted to a final concentration of 0.5 μg/μL with HPLC grade water containing 0.1% (v/v) formic acid and 3% (v/v) acetonitrile. One millilitre of each sample was injected into an UltiMate® 3000 rapid separation liquid chromatography (RSLC, Dionex Corporation, Sunnyvale, CA) coupled online to a Q Exactive HF Hybrid Quadrupole‐Orbitrap mass spectrometer (ThermoFisher Scientific, Waltham, MA). Peptides were eluted over a 90‐min gradient, following previously described LC/MS methodology.^22^ Details of the methods are described in the Supplementary materials.

### 2.5 Data analysis and protein quantification

Data analysis was carried out using MaxQuant version 1.6.1.0. (Max Planck Institute for Biochemistry, Munich, Germany). Peptide MS/MS database search was applied against UniProtKB human proteome containing 74 788 proteins (May 2017; http://www.uniprot.org/), in addition to BSA (internal standard). Label‐free absolute protein quantification was executed using the Hi‐N method.^23^ A list of detected DMETs and peptide sequences used for their quantification is provided in Table S3, with additional details in the Supplementary materials. The calculated abundances were expressed in units of pmol/g of mucosal tissue. This was done by scaling up protein concentrations in homogenates with mucosal weight (in g) prepared for homogenization of each sample.

### 2.6 DMETs' absolute abundance comparison among sample groups

Absolute abundances of ileum and colon DMETs in I‐CD and HN‐CD samples were compared with healthy DMET abundances. Differences of ≥2‐fold between the groups were considered as disease‐related changes.

### 2.7 Statistical data analysis

Statistical data analyses were carried out using GraphPad Prism version 8 (La Jola, CA, USA) and R v3.6.1. The data followed a non‐normal distribution (as determined by the Shapiro–Wilk normality test), and hence non‐parametric statistics were used. Expression data were presented as mean ± standard deviation (SD), and coefficient of variation (CV) was used for inter‐individual variability in abundance. Statistical differences in abundance were assessed using the Mann–Whitney U‐test for each segment (ileum or colon). Statistical significance employed a cut‐off *P*‐value of 0.05. Principal components analysis (PCA), utilizing percentage identical peptide (PIP) and percentage identical protein (PIPr), calculated as previously described,^2^, ^19^ was used to assess biological and technical variation within the groups.

### 2.8 Assessment of technical and analytical variability

To assess technical variability, six samples, representing all groups of ileum and colon, were prepared in triplicate, and analysed under the same LC–MS/MS conditions. For the assessment of between‐ and within‐batch variability, a pool of healthy colon samples (*n* = 5) was prepared once and analysed at the start and end of each batch run (eight runs). %CV was used to assess technical and analytical variability between replicates. The lower limit of quantification (LLOQ) was estimated based on the resultant CV for ileum and colon targets.

### 2.9 Physiologically based pharmacokinetic (PBPK) simulations

The utility of the generated abundance data in predicting the disposition of oral drugs in CD was assessed using a PBPK model in the Simcyp® Simulator V19 (Certara, Sheffield, UK). PBPK simulations were performed using Sim‐Healthy Volunteers virtual population and a recently created active CD population.^5^ The best practices related to generating the population files were followed according to guidelines by Curry et al.^24^

DMET abundance in I‐CD and HN‐CD groups were incorporated (as applicable) in the active CD population, assuming the intestine absorption and metabolism capacity in CD patients is between the inflamed and non‐inflamed states. These changes were implemented in the simulator based on the relative changes of DMETs between healthy and I‐CD or HN‐CD samples. Similarly, the relative changes of the CV values were assigned in each population file. Details of the incorporated abundance values and their CVs in I‐CD and HN‐CD tissues for the enzymes/transporters relevant to the oral drugs simulated in this study are presented in Table S4.

Twelve drugs were selected for this study, primarily based on the availability of a mechanistic absorption model rather than a single compartmental absorption rate (ka). These drugs were under either Dissolution, Absorption, and Metabolism (ADAM) sub‐models or Multilayer‐ADAM (M‐ADAM) sub‐model for the substrate. Thus, the settings allowed the investigation of the impact of changes in the intestine DMETs proteomic profile in CD and associated physiological variability. The ADAM absorption model accounts for the intestine upper (duodenum, jejunum 1&2 and ileum 1–4) and lower (colon) segments' anatomical and physiological characteristics (transit time, pH, blood flows, etc.). In this model, absorption and metabolism of drugs is conducted in the enterocyte compartment, where metabolizing enzymes and transporters are distributed based on their abundance in each segment.^25^, ^26^ Two drug files (budesonide and midazolam) were created,^5^ with the M‐ADAM model, with clinical systemic exposure data from both CD population^27^, ^28^ and healthy volunteers.^27^, ^29^ These drugs were chosen because of availability of PK clinical data in CD and healthy subjects, where significant alteration is reported. Their relevance to CD was also a key factor: budesonide is used to control CD and midazolam used before endoscopy procedures. Additionally, previously conducted predictions^5^ were available to enable the investigation of the utility of the generated DMETs proteomics data. Ten drugs were selected from the Simcyp substrates and inhibitors library built using the ADAM model. Since the number of ADAM model drugs in the Simcyp library is limited, all available drugs were selected to increase the number of possible parameters investigated (different DMETs substrates, extraction ratio, blood protein affinity).

In addition to the primary selection criteria for simulation, many of these drugs (verapamil, statins, valsartan, digoxin, dabigatran, gemfibrozil and celecoxib) are potential candidates to be used in CD patients to control comorbidities/conditions (cardiovascular diseases, metabolic disorders, hypertension and pain management) strongly association with CD.^30^, ^31^ Details of the drugs, their protein binding, formulation and enzymes and/or transporters relevant to their PK and previous performance verifications are shown in Table 1. Simulations were conducted using four models as follows:

**Model 1 (M‐1):** CD population with intestine DMET abundance data from I‐CD tissues and normal albumin level.
**Model 2 (M‐2):** CD population with intestine DMET abundance data from I‐CD tissues and reduced albumin level.
**Model 3 (M‐3):** CD population with intestine DMET abundance data from HN‐CD tissues and normal albumin level.
**Model 4 (M‐4):** CD population with intestine DMET abundance data from HN‐CD tissues and reduced albumin level.

**TABLE 1:** Oral drugs used for PBPK simulations with active CD population. The drugs were from Simcyp V19 library with ADAM absorption model or created (midazolam and budesonide drug files) with M‐ADAM absorption model.

Drug	Oral formulation	Intestine metabolizing enzyme/transporter^a^	Binding to blood proteins (albumin or α1‐AGP)^b^	Performance verification
Budesonide	Controlled release solid formulation	CYP3A4, CYP2C9^32^ and P‐gp^33^	80–90% bound to albumin^34^	Figure S1 and Alrubia et al. (2022)^5^
Celecoxib	Solution	CYP2C9 and CYP3A4	~95% bound to albumin^35^	Kilford et al. (2022) Figure 3 and Table S1, ^36^
Crizotinib	Immediate release solid formulation	CYP3A4 and P‐gp	~91% bound to albumin^37^	Chen et al. (2022) Figure 1 and Table 2^38^
Digoxin	Solution	P‐gp	~25% bound to albumin^39^	Cilliers et al. (2024) Figure S1 and Table S2, ^40^
Dabigatran etexilate	Solution	CES1, CES2^41^ and P‐gp	~35% bound to albumin^42^	El‐Khateeb et al. (2021) Figure 6^43^
Gemfibrozil	Solution	UGT2B7^44^	~98% bound to albumin^45^	Kilford et al. (2022) Table S1, ^36^
Midazolam	Solution	CYP3A4 and CYP3A5^32^	~95% bound to albumin^46^	Figure S2 and Alrubia et al. (2022)^5^
Pravastatin	Solution	MRP2^47^	~50% bound to albumin^48^	Hartauer et al. (2024) Figure 2, ^49^
Ritonavir	Immediate release solid formulation	CYP2D6, CYP3A4 and CYP3A5	~99% bound to α1‐AGP^50^	Kilford et al. (2022) Table S1, ^36^
Rosuvastatin	Solution	BCRP and OATP2B1	~90% bound to albumin^51^	Bowman et al. (2021) Figure 2 and Table S2, ^52^
Valsartan	Immediate release solid formulation	CYP2C9 and MRP2	~95% bound to albumin^53^	Figure S3, provided by Simcyp Certara
Verapamil	Solution	CYP2C8, CYP3A4, CYP3A5, P‐gp and MRP2	~90% bound to albumin^54^	Kilford et al. (2022) Table S1, ^36^

Abbreviation: α1‐AGP, alpha‐1‐acid glycoprotein.

^a^ Based on the incorporated enzymes and transporters in Simcyp drug file.

^b^ The protein listed is based on what Simcyp drug profile used as the primary protein binding to the drug.

For each model and drug, the trial design had: 10 trials with 10 virtual individuals (100 virtual subjects), female‐to‐male ratio 50/50, age range 18‐65 years, and study duration 24 h under fasted conditions. The effects of abundance changes and other systems parameters in CD on oral drug exposure were assessed by comparing mean systemic exposure parameters (area under the curve [AUC_0‐24h_] and maximum plasma concentration [*C*_max_]) from the plotted mean systemic concentration (Csys)–time profiles. Only budesonide and midazolam had a trial design that matched the clinical trial specifications (Table S5).

### 2.10 Nomenclature of targets and ligands

Key protein targets and ligands in this article are hyperlinked to corresponding entries in http://www.guidetopharmacology.org, and are permanently archived in the Concise Guide to PHARMACOLOGY 2019/20.^55^, ^56^

## 3 RESULTS

### 3.1 Assessment of technical and analytical variability

Technical replicates (three per sample) were run for three ileum and three colon samples. We were interested in all the detected targets related to drug metabolism and disposition, listed in Table S3. Technical variability in abundance data was within 20% (%CV) for 84% and 71% of targets for ileum and colon, respectively (Figure S4). Sixteen targets reflected the highest variability (>20%CV) in ileum, of which three were not consistently detected. Additionally, 24 targets showed variability of >20%CV in the colon, of which seven were not consistently detected.

Based on a cut‐off technical variability of 20% in quality control samples, the LLOQ for ileum targets was 0.11 pmol/mg protein, which is around 1.22 pmol/g of mucosal tissue. LLOQ for colon was 0.2 pmol/mg protein, which is around 1.88 pmol/g of mucosal tissue. Batch‐to‐batch analytical variability was within 20% for 75% of targets.

### 3.2 Ileum DMET absolute abundance in Crohn's samples compared with healthy controls

**FIGURE 1:**
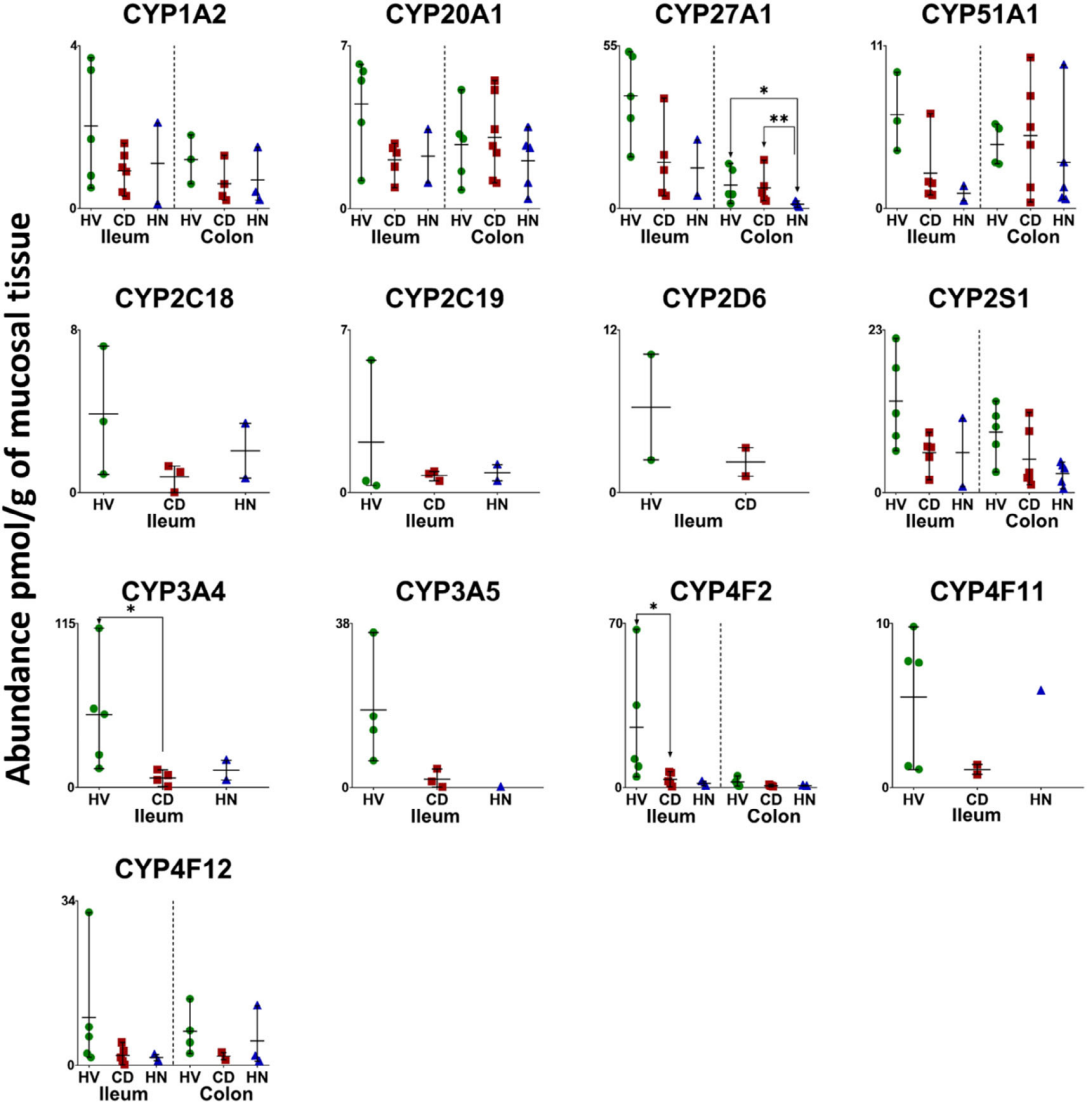
Individual absolute abundance of cytochrome P450 enzymes (CYPs) in pmol/g of mucosal tissue of healthy, inflamed and non‐inflamed Crohn's disease ileum and colon samples (HV: healthy volunteers; CD: inflamed Crohn's disease and HN: histologically normal Crohn's disease). Horizontal lines represent means and bars represent maximum and minimum values. Asterisks (*) represent statistical significance (a post‐hoc Mann–Whitney test, *P* < 0.05) (*P = <0.05 and **P < 0.0087) comparisons between inflamed, non‐inflamed and healthy.

**FIGURE 2:**
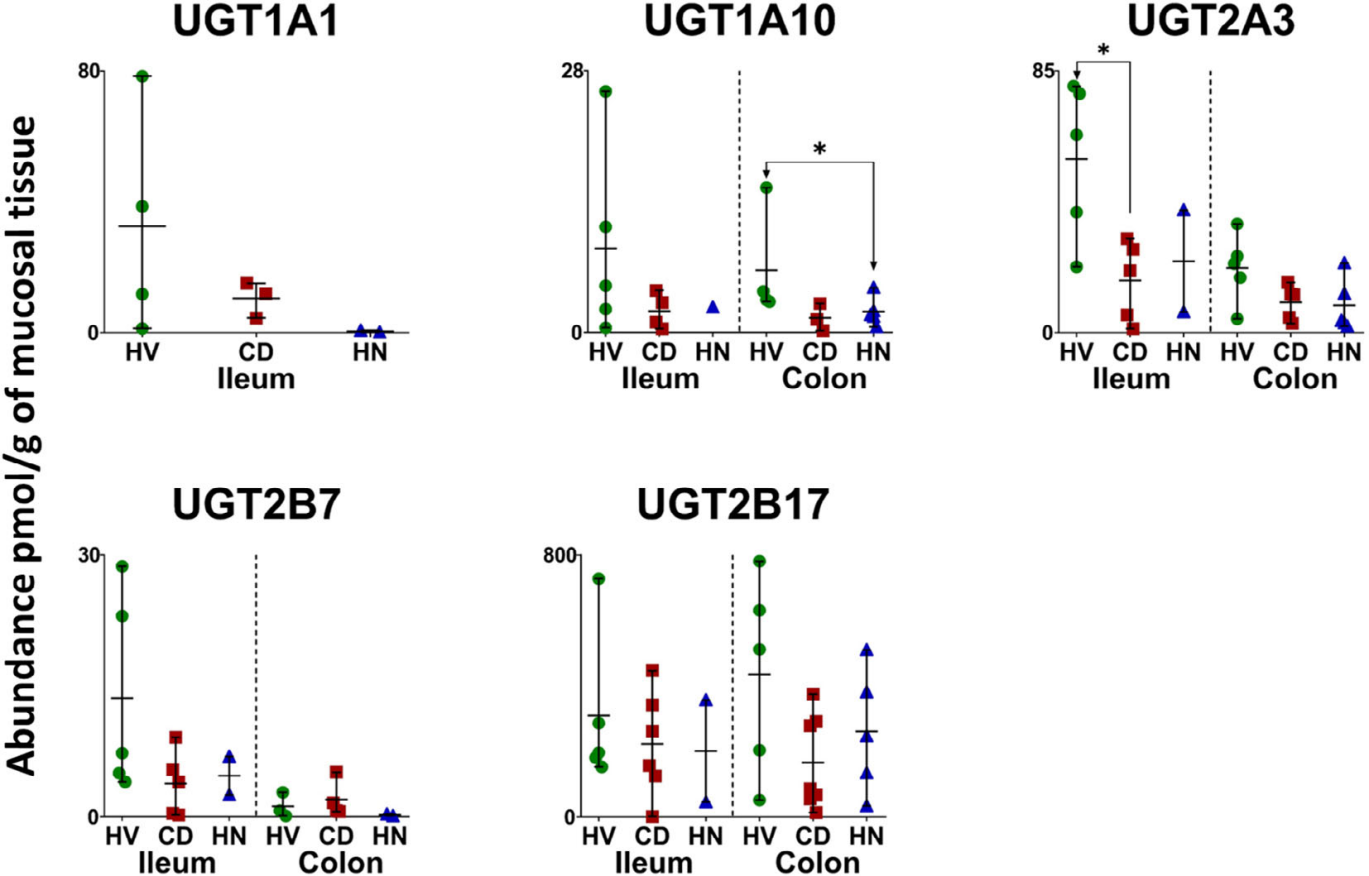
Individual absolute abundance of uridine‐5’‐diphospho‐glucuronosyltransferase enzymes (UGTs) in pmol/g of mucosal tissue from healthy, inflamed and non‐inflamed Crohn's disease ileum and colon samples (HV: healthy volunteers; CD: inflamed Crohn's disease and HN: histologically normal Crohn's disease). Horizontal lines represent means and bars represent maximum and minimum values. Asterisks represent statistical significance (Mann–Whitney test, *P < 0.05) for comparisons of inflamed and non‐inflamed with healthy tissue.

**FIGURE 3:**
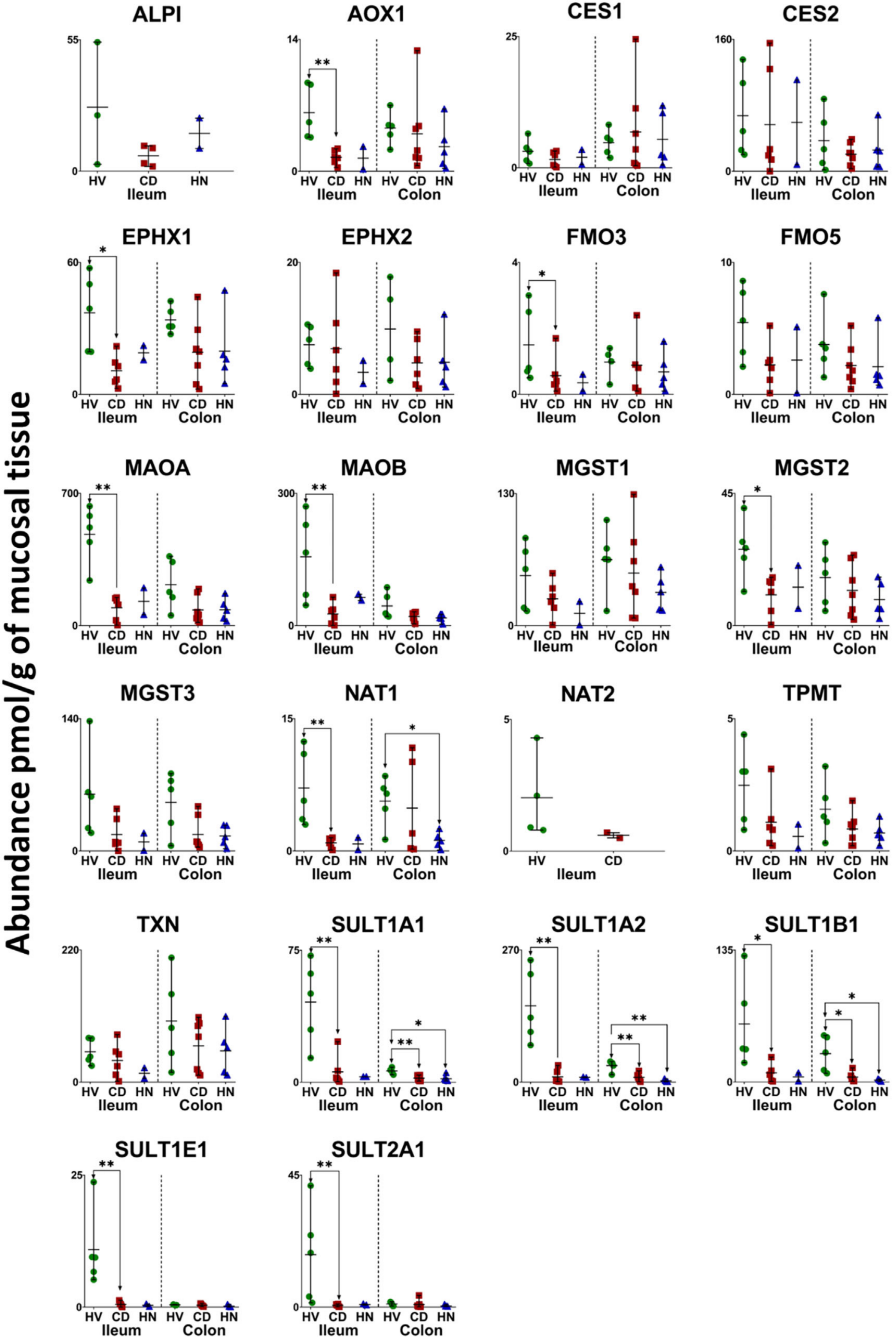
Individual absolute abundance of non‐CYP/non‐UGT enzymes in pmol/g of mucosal tissue from healthy, inflamed and non‐inflamed Crohn's disease ileum and colon samples (HV: healthy volunteers; CD: inflamed Crohn's disease and HN: histologically normal Crohn's disease). Horizontal lines represent means and bars represent maximum and minimum values. Asterisks represent statistical significance (Mann–Whitney test, *P = <0.05 and **P < 0.0087) for comparisons of inflamed and non‐inflamed with healthy tissue.

**FIGURE 4:**
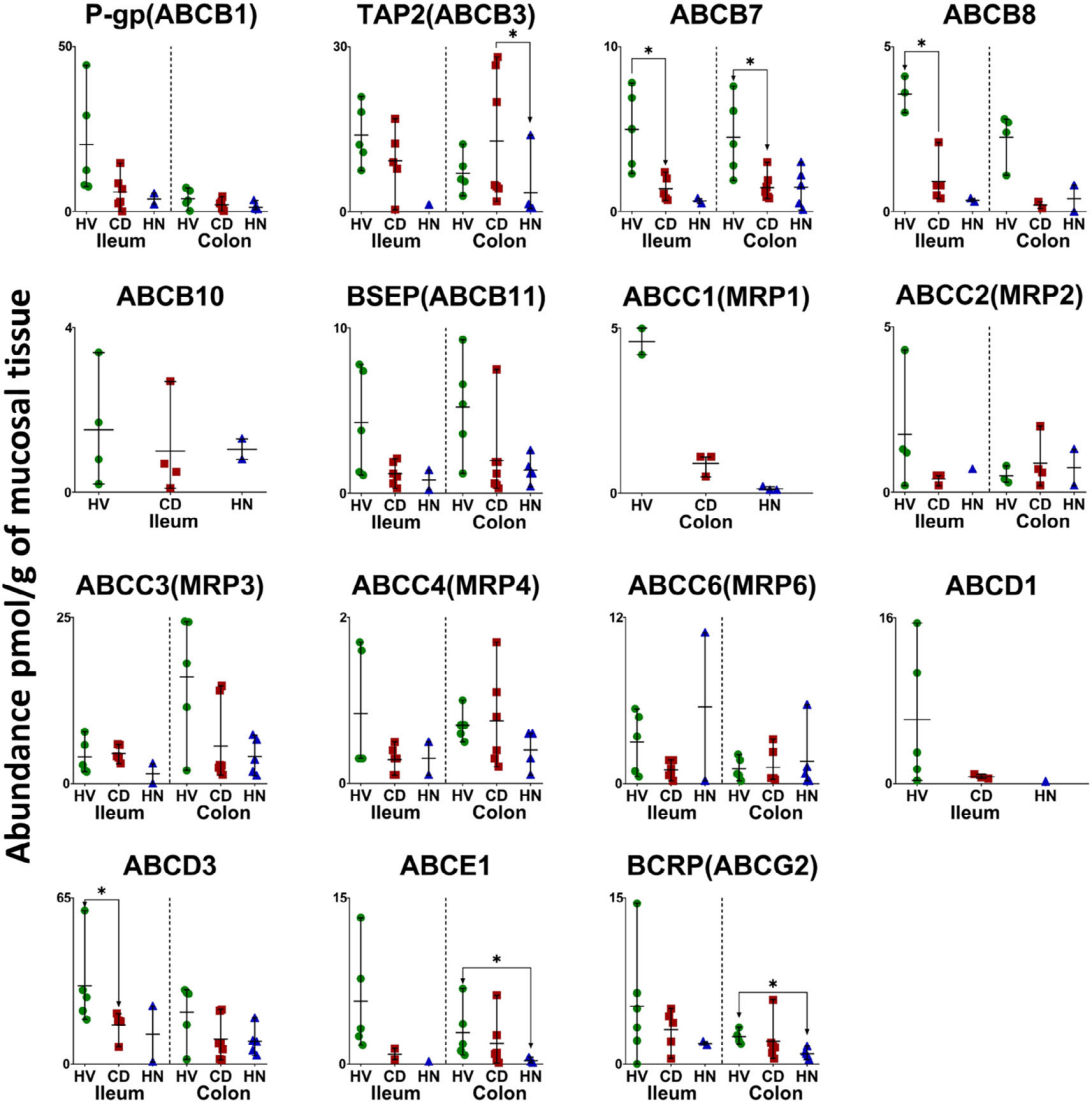
Individual absolute abundance of ATP‐binding cassette (ABC) transporters in pmol/g of mucosal tissue from healthy, inflamed and non‐inflamed Crohn's disease ileum and colon samples (HV: healthy volunteers; CD: inflamed Crohn's disease and HN: histologically normal Crohn's disease). Horizontal lines represent means and bars represent maximum and minimum values. Asterisks represent statistical significance (Mann–Whitney test, *P < 0.05) for comparisons between inflamed, non‐inflamed and healthy tissue.

**FIGURE 5:**
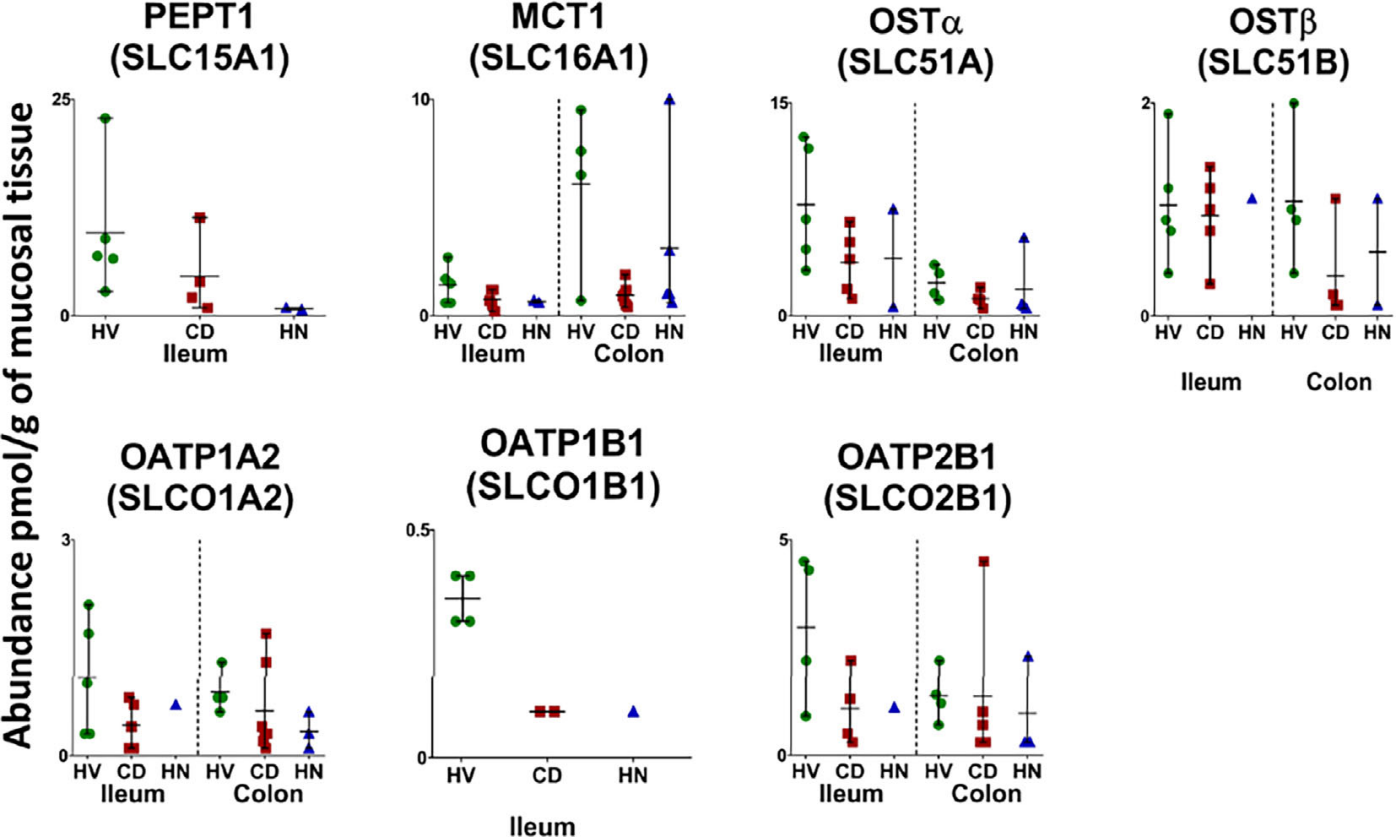
Individual absolute abundance of solute carriers (SLC) in pmol/g of mucosal tissue from healthy, inflamed and non‐inflamed Crohn's disease ileum and colon samples (HV: healthy volunteers; CD: inflamed Crohn's disease and HN: histologically normal Crohn's disease). Horizontal lines represent means and bars represent maximum and minimum values. Statistical significance (Mann–Whitney test, *P* < 0.05).

In inflamed and healthy samples, 13 CYPs, 5 UGTs, 22 non‐CYP/non‐UGT enzymes, 14 ABC and 7 SLC transporters were quantified. The same proteins were quantified in the HN‐CD group except that CYP2S1 and NAT2 were not detected. Figures 1, 2, 3, 4, 5 show the abundance of quantified DMETs. A summary of mean values (pmol/g of mucosal tissue) in each group is presented in Table S6.

I‐CD showed significant reduction in the expression of CYP3A4, CYP4F2, UGT2A3, 12 non‐CYP/non‐UGT enzymes (Figures 1, 2 and 3), ABCB7, ABCB8 and ABCD3 (Figure 4) relative to healthy samples. Fold changes are presented in Figures S5 and S6, ranging from 2‐fold (CES1, ABCD3 and BCRP ^57^) to 28.5‐fold (SULT2A1) reduction. Several targets with ≥5‐fold reduction (CYP3A5, CYP4F11, ABCD1 and ABCE1) did not, however, show statistical significance. No targets showed higher expression in I‐CD compared with healthy samples. When I‐CD was compared with HN‐CD, fold reduction ranged from 2.4 (MAOB)^58^ to 5.7 (MRP6) fold, while fold increase ranged from 2 (TPMT) to 28.7 (UGT1A1) fold. The HN‐CD group showed no significant difference across all targets when compared with the healthy and inflamed groups, likely due to small sample size, as only two samples were available. Fold reduction ranged between 2 (CYP20A1, EPHX1, MGST2 and OST‐α ^59^) and 128.3 (CYP3A5) compared with healthy baseline; no increase in expression in disease was observed.

Inter‐individual variability in ileum targets is summarized in Table S6. As only two samples were available in the HN‐CD group, assessment of inter‐individual variation was not possible. In both healthy and inflamed tissue, %CV varied from about 15% to just over 100% for all targets. CYP2C19 showed the highest variation in healthy samples (140%, detected in three samples), while SULT1A1 had the highest variation in I‐CD (146%, detected in all samples). Higher variability in non‐CYP/non‐UGT expression was observed in CD samples compared with healthy ones, as seen with CES2 (76% in healthy and 116% in inflamed samples), EPHX2 (~41% in healthy and ~98% in inflamed samples), SULT1A1 (52% in healthy and 146% in inflamed samples), SULT1A2 (~48% in healthy and ~131% in inflamed samples) and others.

PCA analysis in Figure S7 (ileum) showed that healthy samples form one cluster, while the I‐CD and HN‐CD groups clustered together. One inflamed sample was an outlier; this is the only donor who had a bowel resection before the sample was collected. Either the disease progression or the surgery may have contributed to this observation.

### 3.3 Colon DMET absolute abundance in Crohn's samples compared with healthy controls

In the colon, 7 CYPs, 4 UGTs, 20 non‐CYP/non‐UGT enzymes, 13 ABC transporters and 5 SLCs were compared among inflamed, histologically normal CD and healthy samples. A summary for each group is presented in Table S7. Figures 1, 2, 3, 4, 5 show the abundance of CYP, UGT, non‐CYP/non‐UGT, ABC and SLC targets, respectively.

Similar to the ileum, CD led to reduced expression of ADME proteins in the colon. Notable examples include a 2‐fold reduction in CYP1A2, TPMT, P‐gp and OST‐α and a 10‐fold reduction in ABCB8 in I‐CD. Multiple targets showed apparent reduction but did not reach statistical significance, probably owing to the small number of samples and high inter‐individual variation. Interestingly, several proteins (CYP27A1, UGT2B7, NAT1,^60^ SULT1A2, SULT2A1, SULT1B1, TAP2, MRP1,^47^ MRP4, ABCE1 and BCRP) exhibited higher expression in I‐CD than in HN‐CD. Fold changes among the colon CD groups and the healthy cohort are presented in Figures S8 and S9.

Assessment of inter‐individual variability in each group (Table S7) showed that %CV ranged from 11% to ~169% for all targets. In healthy samples, the highest variation was observed with UGT2B7 (detected in three samples) at ~118%. SULT2A1 recorded the highest variation in I‐CD samples (detected in all samples) at ~157%, while TAP2 had the highest variation in the HN‐CD group (detected in all samples) at ~169%. A wider range of abundance variation among the CD colon sample groups compared with healthy samples was reflected across most DMETs. The highest variability was seen with non‐CYP/non‐UGT enzymes and transporters. Non‐CYP/non‐UGT enzymes variability ranged from ~18% to 97% (EPHX1 and CES2, respectively) in healthy and from ~51% to 157% (MAOB and SULT2A1, respectively) in CD (inflamed and histologically normal) samples. ABC transporter variability in healthy samples ranged from ~11% to 87% (MRP1 and ABCE1, respectively), and in CD between ~39% and 169% (MRP1 and TAP2, respectively). For SLCs, variability ranged from ~36% to 63% (OATP1A2 and OST‐β,^59^ respectively) in healthy samples and from ~52% to135% (OST‐α and OST‐β, respectively) in CD. This observation indicates higher variability in disease. PCA analysis (Figure S10, colon) showed that, similar to the ileum, I‐CD and HN‐CD samples cluster together with healthy samples slightly separated.

### 3.4 Impact of Crohn's disease‐related systems changes and altered intestine DMET abundance on oral drug pharmacokinetics

**FIGURE 6:**
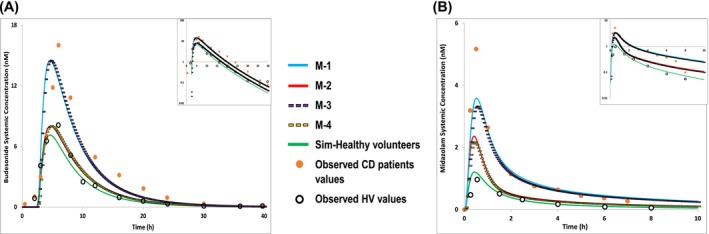
Simulated mean plasma concentration–time profiles of **(A)** budesonide and **(B)** midazolam using a CD population with metabolizing enzymes and transporters abundance values in Crohn's disease generated in this study and other systems changes^5^ (**M‐1**; intestine DMET abundance data from I‐CD tissues and normal albumin level, **M‐2**; intestine DMET abundance data from I‐CD tissues and reduced albumin level, **M‐3**; intestine DMET abundance data from HN‐CD tissues and normal albumin level, **M‐4**; intestine DMET abundance data from HN‐CD tissues and reduced albumin level; and Simcyp V19 default healthy population, with normal albumin levels) compared with the mean observed in‐vivo data, midazolam (*n* = 8) and budesonide (*n* = 6),in Crohn's disease patients^27^, ^28^ and healthy volunteers (midazolam *n* = 16 and budesonide *n* = 8)^27^, ^29^ (symbols).

Four CD models were used (see Methods section). Midazolam clinical data were derived from active CD patients, while budesonide data were from a mixed active and inactive CD population. The clinical AUC and *C*_max_ values for budesonide were very close to those predicted by M‐1 and M‐3 (Figure 6A and Table S8), where CYP3A4 and P‐gp abundances are reduced and albumin levels are normal. The predicted/observed ratios were 0.92 for AUC_0‐∞_ and 1.04 for *C*_max_. For midazolam, the best fit was M‐1, where CYP3A4 abundance is reduced and albumin levels are normal. The predicted/observed ratio was 0.61 for AUC_0‐∞_ with 0.44 for *C*_max_ (Figure 6B and Table S9). The 95% confidence interval (CI) of the simulated concentration–time profiles of midazolam and budesonide, compared to their mean observed clinical data in CD patients, are presented in Figure S11.

**FIGURE 7:**
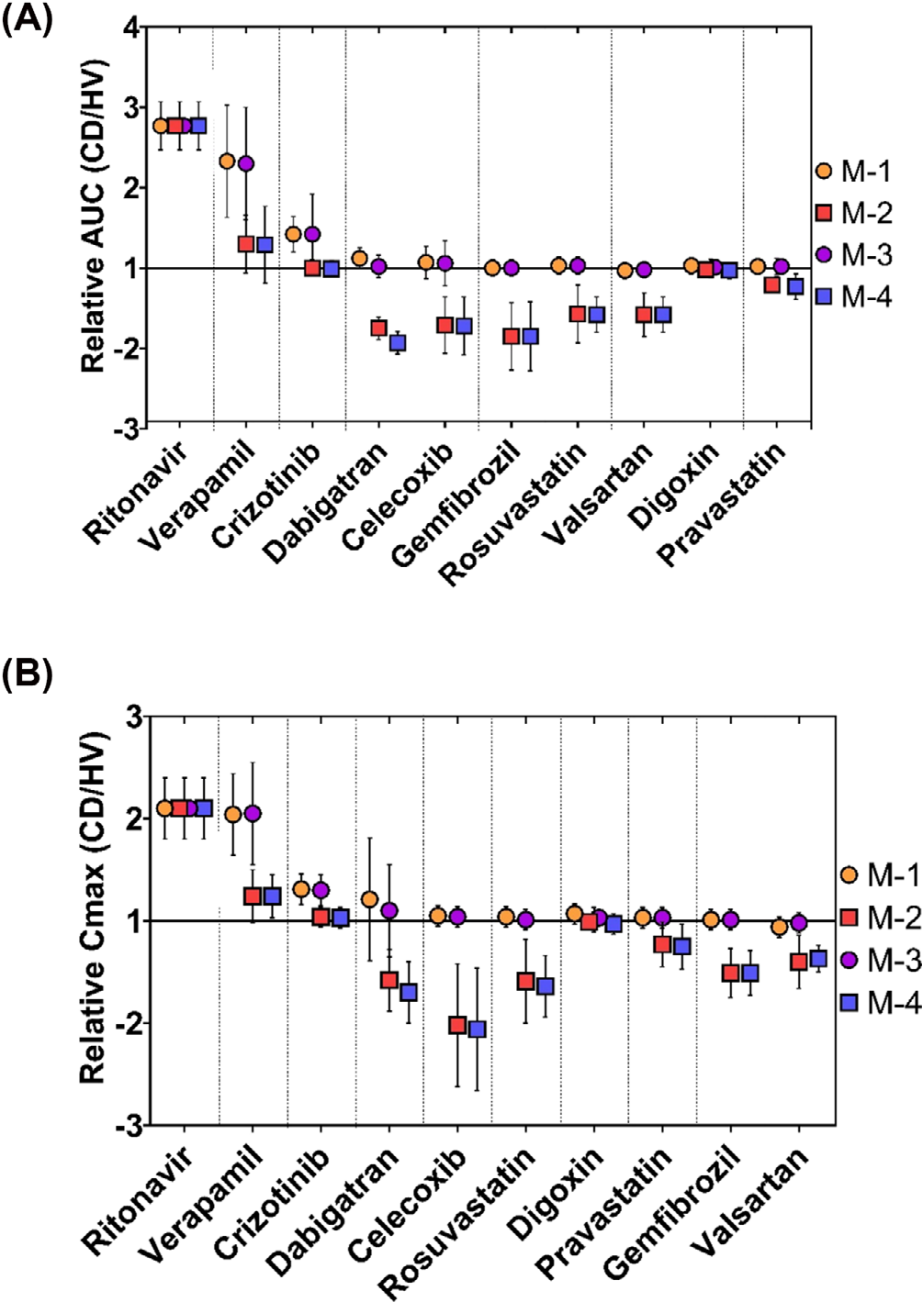
Simulated relative **(A)** AUC and **(B)** *C*_max_ between Crohn's disease (CD) and healthy (HV) populations for 10 oral substrates. The symbols represent the mean relative values, and the lines represent the standard deviation (SD). The applied Crohn's population models: **M‐1** (intestine DMET abundance data from I‐CD tissues and normal albumin level), **M‐2 (**intestine DMET abundance data from I‐CD tissues and reduced albumin level**), M‐3** (intestine DMET abundance data from HN‐CD tissues and normal albumin level) and **M‐4** (intestine DMET abundance data from HN‐CD tissues and reduced albumin level).

The predicted AUC and *C*_max_ for the 10 drugs selected from the Simcyp library, based on simulations with the Simcyp healthy population and CD population modified with the DMET changes from the current study (M‐1 to M‐4), are shown in Figure 7. Crohn's disease led to a >2‐fold increase in AUC for ritonavir (2.8) and verapamil (2.3) and in *C*_max_ (2.1 for both). However, dabigatran showed a reduction in AUC and *C*_max_ (1.9‐ and 1.7‐fold, respectively), as did gemfibrozil (1.85‐ and 1.5‐fold, respectively) and celecoxib (1.7‐ and 2.1‐fold, respectively). For the other drugs, AUC and *C*_max_ relative change to healthy subjects varied between the four models but did not exceed 1.7‐fold.

## 4 DISCUSSION

CD patients may be exposed to several oral drugs owing to the chronic and early onset of the disease and the commonly associated comorbidities such as cardiovascular diseases, metabolic syndromes, hypertension, neuropsychological disorders and liver and GI‐related diseases.^31^, ^61^ Additionally, CD might be a comorbidity in patients treated for other unrelated health issues. Inflammation caused by CD can alter expression of DMETs in affected and adjacent tissue, and may induce systemic responses, such as liver injury.^53^, ^54^, ^57^, ^58^, ^59^, ^60^, ^61^, ^62^, ^63^ Quantitative data on DMETs expression in CD are limited. This study is the first to measure absolute abundance of DMETs in ileum and colon of individual CD patients to enable more reliable predictive PBPK models of the fate of oral drugs in CD populations.

While guidelines exist for the assessment of potential pharmacokinetic changes in patients with specific organ impairment, there is no guidance encouraging such studies in CD patients. The latest US FDA guidance encourages conduction of clinical trials in more diverse cohorts of patients,^6^ with such inclusive trials expected to take time before implementation. The absence of guidance can create uncertainty in dosing drugs in patients with CD as a comorbidity, and prescribers may assume lack of change in drug response. Moreover, a meta‐analysis of existing cases failed to provide any trends in observed changes that can be linked to simple characteristics, such as drug solubility or affinity to enzymes, mainly owing to the multifactorial nature of changes that affect one enzyme substrate more than another when affinity was similar.^5^

The active phase generally exhibits more aggressive alterations that may affect oral drug disposition.^64^, ^65^, ^66^, ^67^, ^68^, ^69^, ^70^ For DEMTs expression, both active and inactive phases of CD have been associated with alteration of DMETs. Generally, DMETs expression levels showed more pronounced alteration in active CD patients.^5^ We worked with surgical samples and so patients were necessarily those who did not achieve remission purely by pharmacotherapy. Thus, the active disease state of the patients (based on persistence of symptoms) is most likely the cause of the reported DMETs alterations. The literature suffers from lack of distinction between changes in DMETs expression in active and inactive CD.^5^ Where there are published studies, comparison of DMETs expression based on CD phases is limited to one study, where BCRP, ASBT ^71^ and SULT2A1 showed higher alteration in active CD.^13^

Notably in this study, CYP3A4, responsible for metabolizing about 30% of clinical drugs,^72^ showed significant downregulation of its ileum expression (~8 and ~4‐fold in I‐CD and HN‐CD, respectively) relative to healthy subjects. The CYP3A family represents approximately 80% of intestinal CYP enzymes where CYP3A4 is the most abundant.^73^ Inflammatory cytokines (TNF‐α, INF‐γ IL‐1β and IL‐6) are upregulated in CD^74^ and linked to suppression of CYP3A expression.^75^, ^76^ Other CYPs can be downregulated by similar mechanisms.^10^ Pregnane X receptor (PXR),^77^ which regulates CYP3A expression, is dysregulated in inflammatory conditions.^78^, ^79^ This dysregulation may contribute to the observed CYP3A suppression. The observed trend is in line with previous gene, relative and protein expression data, with varying levels of reduction.^14^, ^19^, ^80^, ^81^ Drug–disease interaction studies have also reported a significant alteration in the PK of CYP3A4 oral substrates in CD patients.^27^, ^28^, ^82^ Up to 62% of inflammatory bowel disease (IBD) patients receive oral glucocorticoids (prednisone and budesonide; CYP3A4 substrates)^83^, ^84^ within 10 years of diagnosis.^85^ Treatment failure is reported in up to 47% of patients with active CD.^4^ The downregulation of phase II metabolizing enzymes can be similarly explained. Elevated inflammatory cytokines have been linked to reduced mRNA expression and enzymatic activity of several UGTs and SULTs.^86^, ^87^ This response is also linked to the dysregulation of their relevant nuclear receptors and transcription factors in other inflammatory conditions.^86^, ^88^, ^89^, ^90^ However, information on their expression in IBD populations is limited.^5^, ^91^ We observed a significant reduction in colon UGT1A10^44^ (up to ~4‐fold), consistent with previously reported data in pooled samples.^19^ UGT1A10 is involved in the metabolism of oral anticancer and angiotensin receptor blockers.^92^, ^93^ SULTs were also significantly reduced in both ileum and colon from inflamed and non‐inflamed sources. mRNA expression of SULT1A2 and SULT2A1 was significantly reduced in ileum and colon from CD patients.^13^, ^19^, ^81^ Protein expression of SULT1A2 and SULT2B1 were reduced in pooled samples.^19^ These enzymes catalyse sulfate conjugation in various oral substrates, including paracetamol and minoxidil.^94^, ^95^ Alteration in other UGTs and SULTs were reported,^19^, ^81^ but these were either below the LOQ in our samples or not significantly changed.

Other non‐CYP/non‐UGT enzymes such as AOX1 ^96^ and FMOs showed significant expression reduction, not previously reported in CD patients. FMOs metabolize oral chlorpromazine, promethazine, brompheniramine, cimetidine, ranitidine and itopride.^97^ AOX1 metabolizes oral immunosuppressant drugs, methotrexate and azathioprine, prescribed to control refractory CD.^98^, ^99^, ^100^ Azathioprine is further metabolized by TPMT, which was reduced by ~4.5‐fold in HN‐CD ileum samples. TPMT deficiency and genetic variation can cause serious toxicity related to accumulation of azathioprine and its metabolite.^101^, ^102^ Around 17% of IBD patients may not tolerate these drugs, potentially requiring dose adjustment.^103^ MAOA,^104^ MAOB and NAT1 expression changes were reported in CD pooled samples, with MAOA showing higher expression relative to control.^19^ Here, MAOA and MAOB were significantly reduced in CD ileum (up to ~6‐fold). MAOA and MAOB catalyse amine neurotransmitters degradation and/or neuromodulators.^105^ MAO inhibitors are sometimes given by non‐oral routes (selegiline) or as pro‐drugs (ladostigil) to avoid interaction with intestinal and hepatic MAOs.^106^, ^107^, ^108^ NAT1 was significantly decreased in CD ileum (up to 9‐fold) and in HN‐CD colon (~5‐fold). NAT1 metabolizes oral aminosalicylate, which is used to control CD. A systematic review found aminosalicylate was not more effective than placebo.^109^ Mesalamine exhibited higher systemic exposure in CD patients compared with healthy volunteers and ulcerative colitis patients,^110^ but lower bioavailability in CD patients in remission compared with healthy subjects.^111^

A reduction (≥5‐fold) was observed for P‐gp and PEPT1 ^112^ in HN‐CD ileum samples and for MRP1, BCRP and MCT1 ^113^ in CD colon samples. Literature on CD patients focused on P‐gp because of its relevance to various drugs.^114^, ^115^ P‐gp mRNA showed a significant reduction in CD ileum and colon.^14^, ^16^, ^116^ BCRP mRNA decreased in CD ileum,^13^, ^19^ a major multidrug‐resistance contributor for oral chemotherapy.^117^ MCT1, a versatile acid transporter,^118^ also decreased in CD colon and ileum.^19^, ^119^ PEPT1 mRNA expression was reported to be upregulated,^15^ while its protein expression was downregulated.^19^ Oral β‐lactam antibiotics and ACE inhibitors are substrates of PEPT1.^120^ Alterations in these transporters are linked to inflammatory mediators (ILs, TNF‐α and INF‐γ).^12^, ^119^, ^121^ While most studies reported downregulation of intestinal and hepatic transporters with inflammation, increased expression of P‐gp and MRP3 ^47^ was also reported.^12^, ^76^ Moreover, mRNA expression of ABC transporters was found to correlate with PXR activation, which impacts intestinal epithelial barrier function, hence intestine permeability.^122^ In contrast to direct correlation between inflammation severity and altered abundance,^80^, ^119^ this study found that several targets were more significantly reduced in non‐inflamed relative to inflamed tissue. This indicates that the inflammation impact is not localized. In a study comparing CYP3A4, 3A5 and P‐gp gene expression in CD inflamed and non‐inflamed tissues, only CYP3A4 showed a significantly lower expression in inflamed tissue.^80^ OATP2B1 and 4A1 mRNA expression displayed no difference between inflamed and non‐inflamed ileum. However, since non‐inflamed tissue is adjacent to inflamed tissue, this limits generalization of the findings to upper intestine segments in CD patients. In addition, the temporal disconnect between mRNA and actual protein levels should be considered due to faster elimination of mRNA compared with actual protein, as discussed in the context of liquid biopsy assays where plasma exosomal mRNA has longer elimination half‐lives than tissue mRNA.^123^

Inter‐individual variability of DMETs can affect individual response to the same medication. Such impact is seen in CD patients receiving non‐oral drugs.^124^, ^125^, ^126^, ^127^ For oral azathioprine and mercaptopurine (TPMT and AOX1 substrates), response and toxicity were evaluated due to variation in thiopurine metabolism in IBD patients. Dosage adjustment improved the response and decreased adverse events.^128^ Our data showed that AOX1 abundance variation (up to 106%) and TMPT (up to 100%) were higher in CD than healthy samples. PK of oral budesonide exhibited variability in CD patients receiving the same formulation.^27^, ^129^, ^130^ Budesonide and prednisolone (CYP3A4 and P‐gp substrates) showed high variability in response and adverse events in CD.^4^, ^131^, ^132^ CYP3A4 and P‐gp abundance in our samples showed relatively similar variation in CD and healthy samples. Previously, significant inter‐individual variation of CYP3A4 and P‐gp was reported in ileal and colon tissues of CD patients.^14^

The clinical exposure to CYP3A substrates, midazolam and budesonide, in CD patients was simulated using the collected data. Simulation with the M‐1 model showed improved prediction of midazolam AUC and *C*_max_ from our previous model,^5^ which incorporated relative abundance data from the literature, (predicted/observed AUC from 0.36 to 0.61, and predicted/observed *C*_max_ from 0.3 to 0.44 in the current study). This improvement indicates a strong dependence on CYP3A4 expression and other physiological parameters. *C*_max_ underestimation can mostly be linked to its solution formulation as it tends to be absorbed from the intestine upper segments (duodenum and jejunum), where also the majority of the CYP3A iso‐enzymes are extensively expressed.^133^ Lack of information on the expression of CYP3A4 in these segments can be a major contributor in this underestimation. In addition, gastric emptying time, intestine transit time and permeability play a key role. The gastric emptying and intestine transit times are captured in our model,^5^ yet intestine permeability is a major aspect that needs to be considered to improve the model prediction. Permeability is reported to be altered in response to CD inflammation.^134^, ^135^ This is specifically relevant here as all the participating CD patients in the study are in the active CD phase. For budesonide, the M‐1 model best matched the observed PK data, consistent with previous prediction using literature expression data on CYP3A4, CYP2C9 and P‐gp.^5^ This confirms previous conclusions that budesonide bioavailability alteration in CD patients is mainly due to combinations of various altered system parameters over the disease course, especially liver blood flow and its high extraction ratio (E_H_ = 0.9).^34^

Alteration of the drugs' PK metrics (Table S8 and S9) was not different when abundance data from I‐CD samples were applied compared to HN‐CD data (M‐1 and M‐3). While this can mainly be attributed to the small number of ileum HN samples, it demonstrates that non‐inflamed tissue can cause alterations similar to inflamed tissue and should not be considered healthy as seen in previously reported data.^5^ M‐2 and M‐4 models (with low albumin levels) showed poor prediction, their implementation being based on the reported significant drop of albumin level predominantly in active Crohn's patients.^5^ For both clinical studies, the albumin level of the CD participants was not reported. Instead, the activity of disease in the subjects was reported as active or inactive (budesonide) and only active (midazolam) in CD patients (Table S5). This would lead to assuming that active CD patients would suffer from albumin reduction causing the observed PK alteration, which is not captured by M‐2 and M‐4 model predictions and better captured with the normal albumin models (M‐1 and M‐3), emphasizing the importance of evaluating albumin levels in CD populations as one scenario cannot fit all.

The impact of CD on systemic exposure of 10 oral drugs was assessed with simulated AUC and *C*_max_ changes. Two substrates (ritonavir and verapamil) showed >2‐fold change in their AUC in CD relative to healthy population, probably due to downregulated CYP3A4 in liver^5^, ^28^ and intestine, as well as high blood protein binding (Table 1). Although celecoxib and crizotinib are metabolized by CYP3A4 and highly bound to albumin, change in AUC relative to healthy population was <2‐fold. This might be due to their low to intermediate extraction ratios determined in Simcyp to be 0.3 (0.05–0.61) for celecoxib and 0.47 (0.19–0.72) for crizotinib, which is in agreement with reported literature values (celecoxib E_H_ ~ 0.04–0.2 and crizotinib ~0.44).^37^, ^136^ Thus, changes applied in the hepatic blood flow of the CD population model^5^ will not have an impact on its PK profile. Verapamil and crizotinib (<2‐fold) showed higher sensitivity to reduction in DMETs expression (M1 and M3 models), given by the higher AUC and *C*_max_ alterations, than with albumin reduction (M2 and M4 models), probably due to the involvement of efflux transporters (P‐gp and MRP2). In contrast, ritonavir showed similar sensitivity to the albumin and CYP3A4 levels alteration. Three drugs (celecoxib, ritonavir and verapamil) showed >2‐fold change in *C*_max_ in CD. CYP3A4 played a significant role in the disposition of the drugs demonstrating the highest changes in AUC and *C*_max_ relative to HV, where its abundance was reduced in CD intestine and liver. Conversely, compounds that are primarily renally excreted (digoxin, dabigatran, and gemfibrozil)^39^, ^137^, ^138^ and biliary excreted (pravastatin, rosuvastatin and valsartan)^139^, ^140^, ^141^ showed smaller differences, despite their albumin binding affinity. The change in albumin level caused higher alteration in the applied models (M‐2 and M‐4). This is evident as all drugs with high predicted PK alterations are highly bound to albumin as well as substrates of CYP3A4. Interestingly, the low albumin models have showed low relative AUC prediction of celecoxib, dabigatran, gemfibrozil, rosuvastatin and valsartan (1.9–1.6‐fold). Several drug‐specific factors can affect the PK prediction including the enzyme kinetics (Km and *V*_max_), primary clearance organ and hepatic extraction ratio. All the aforementioned drugs except celecoxib are renally and biliary excreted. This results in a higher free drug fraction caused by low albumin, more sensitive to their non‐hepatic clearance, where the function of the clearance organs is not altered in the CD models. In Simcyp, celecoxib has a high affinity to CYP2C9 (Km ~ 2.7. compared to Km ~ 16.7 for CYP3A4), as a higher free drug fraction is susceptible to CYP2C9 hepatic metabolism, which is only altered in the intestine and unchanged in the liver, causing the predicted (~1.7‐fold) low relative AUC. In general, changes in AUC and *C*_max_ were only slightly higher when protein abundance in I‐CD tissue was applied compared with HN‐CD (M‐1 and M‐3).

The applicability of the current model has only been verified using CYP3A4 substrates. Substrates of other enzymes lack available clinical data or lack information of the model system parameters needed to be incorporated to capture their PK profile. Data on DMETs expression/activity of the upper intestine segments and liver, intestine permeability, enterocytes shedding rate and intestine microbiota of CD patients is warranted to enhance the model prediction performance and widen its applicability.

## 5 CONCLUSION

In summary, availability of information on physiological changes in disease and the generated DMET proteomics enabled building a more reliable PBPK CD population model. For active CD, many physiological changes are directly related to the drugs' systemic availability. The improved virtual CD population model has the potential to assist in guiding dose adjustment for oral medications in the absence of dedicated clinical PK studies investigating such differences.

## AUTHOR CONTRIBUTIONS

This study was designed by S.A., B.A., Z.M.A., A.R.‐H. and J.B. S.A. Collected the date and conducted the experiments. S.A., B.A., Z.M.A. contributed new reagents or analytic tools and analysed the data. S.A. wrote the original draft of the manuscript. B.A., Z.M.A., A.R.‐H. and J.B. reviewed and edited the manuscript. Supervision and project administration was provided by A.R.‐H. and J.B.

## CONFLICT OF INTEREST STATEMENT

The PBPK models were provided by Certara. A.R.H. is a part‐time employee and shareholder of Certara Inc. who provide PBPK software to the pharmaceutical industry. The other authors declare no known conflicts of interest.

## Supporting information


**Table S1.** Demographic and clinical details of Crohn's disease (CD) patients.
**Table S2.** Demographic details of healthy subjects.
**Table S3.** High intensity unique peptide sequences assigned to each DMET and used to quantify their abundance in inflamed, histologically normal CD and healthy samples based on HiN label‐free methodology.^3^
**Table S4.** Input abundance values of drug‐metabolizing enzymes and transporters in the Simcyp Simulator for the created active CD population relative to healthy baseline based on data generated in the present study.
**Table S5.** Specifics of the PBPK‐based simulation workflows for budesonide and midazolam implemented in the Simcyp Simulator and their corresponding trial design parameters.
**Table S6.** Abundance (pmol/g of mucosal tissue) of CYP enzymes, UGT enzymes, non‐CYP/non‐UGT enzymes, ABC transporters, SLCs in inflamed Crohn's disease (I‐CD), histologically normal Crohn's disease (HN‐CD) and healthy ileum samples. Data are presented as mean, standard deviation of the mean (SD) and coefficient of variation (%CV).
**Table S7.** Abundance (pmol/g of mucosal tissue) of CYP enzymes, UGT enzymes, non‐CYP/non‐UGT enzymes, ABC transporters, SLCs in inflamed Crohn's disease (I‐CD), histologically normal Crohn's disease (HN‐CD) and healthy colon samples. Data are presented as mean, standard deviation of the mean (SD) and coefficient of variation (%CV).
**Table S8.** Comparison of predicted and observed ^4^ PK parameters and their fold change in active CD populations with the different applied models (M‐1, M‐2, M‐3 and M‐4) of oral budesonide‐controlled release formulation under fed conditions.
**Table S9.** Comparison of predicted and observed^5^ PK parameters and their fold change in active CD populations with the different applied models (M‐1, M‐2, M‐3 and M‐4) of oral midazolam solution formulation under fasted conditions.
**Figure S1.** Prediction of budesonide plasma concentration for healthy subjects after administration of (**A)** systemic 0.5 mg intravenous (IV) dose (*n* = 24) with observed values from Thorsson et al.,^7^ and (**B)** 18 mg oral (PO) solution (*n* = 8) and (**C)** 18 mg oral (PO) solution log scale with observed values from Edsbäcker et al.,^4^ in the fed state.
**Figure S2.** Prediction of midazolam plasma concentration for healthy subjects (*n* = 16) from Hohmann et al.,^8^ after administration of (**A)** systemic 0.001 mg intravenous (IV) dose with observed values and (**B)** 0.003 mg oral (PO) solution and (**C)** 0.003 mg oral (PO) solution log scale with observed values in the fasted state.
**Figure S3.** Performance verification of valsartan drug profile in Simcyp Simulator provided by Simcyp, Certara. Simulated (black line) and observed (data points) indicate plasma concentration–time profiles of valsartan after a single oral dose of 160 mg in Caucasian (A and B) and Japanese (C and D) subjects (10 trials of 15 healthy volunteers or Japanese male subjects, 20–35 years). The grey lines represent the prediction from individual trials. Dashed lines represent the 5th and 95th percentile of the total virtual population. Observed data were extracted from Sunkara et al. ^6^ (B) and (D) show the data plotted with the *y*‐axis on a log scale.
**Figure S4.** Technical variability in (A) ileum and (B) colon DMETs presented as coefficients of variation (%CV) for different targets in a set of three ileum and three colon samples (prepared in triplicate).
**Figure S5.** Relative change in expression of DMEs (CYPs, UGTs, SULTs and other enzymes) in healthy ileum samples (*n* = 5), inflamed CD ileum (*n* = 6) and histologically normal CD ileum (*n* = 2). Change in expression is shown for (A) inflamed relative to healthy, (B) histologically normal relative to healthy and (C) inflamed relative to histologically normal. Only targets with fold change ≥2 are reported.
**Figure S6.** Relative change in expression of drug transporters in healthy ileum samples (*n* = 5), inflamed CD ileum (*n* = 6) and histologically normal CD ileum (*n* = 2). Change in expression is shown for (A) inflamed relative to healthy, (B) histologically normal relative to healthy and (C) inflamed relative to histologically normal. Only targets with fold change ≥2 are reported.
**Figure S7.** Principal components analysis (PCA) for similarity data based on (**A**) percentage identical peptides (PIP) and (**B**) percentage identical proteins (PIPr). Identified peptides and proteins in 13 ileum samples of healthy, inflamed from Crohn's disease (I‐CD) and non‐inflamed from Crohn's disease (HN‐CD) ileum tissues.
**Figure S8.** Relative change in expression of DMEs (CYPs, UGTs, SULTs and other enzymes) in healthy colon individual samples (*n* = 5), inflamed CD colon (*n* = 7) and histologically normal CD colon (*n* = 5). Change in expression is shown for (A) inflamed relative to healthy, (B) histologically normal relative to healthy and (C) inflamed relative to histologically normal. Only targets with fold change ≥2 are reported.
**Figure S9.** Relative change in expression of drug transporters in healthy colon samples (*n* = 5), inflamed CD colon (*n* = 7) and histologically normal CD colon (*n* = 5). Change in expression is shown for (A) inflamed relative to healthy, (B) histologically normal relative to healthy and (C) inflamed relative to histologically normal. Only targets with fold change ≥2 are reported.
**Figure S10.** Principal components analysis (PCA) of similarity data based on (**A**) percentage identical peptides (PIP) and (**B**) percentage identical proteins (PIPr). Identified peptides and proteins in 17 colon samples of healthy, inflamed from Crohn's disease (I‐CD) and non‐inflamed from Crohn's disease (HN‐CD) colon tissues.
**Figure S11.** Simulation of concentration–time profile of midazolam (*n* = 8) following 0.1 mg solution orally in the fasted state^5^ and budesonide (*n* = 6) following 18‐mg controlled‐release capsule orally in the fed state).^4^ Observed data (orange circles) are compared with the 5th and 95th percentile (upper and lower grey lines) of the total virtual population and the mean prediction profile (green central line) generated from physiologically based pharmacokinetic models of active Crohn's disease population created with metabolizing enzymes and transporters abundance values generated in this study and other systems changes^9^ (M‐1; intestine DMET abundance data from I‐CD tissues and normal albumin level, M‐2; intestine DMET abundance data from I‐CD tissues and reduced albumin level, M‐3; intestine DMET abundance data from HN‐CD tissues and normal albumin level and M‐4; intestine DMET abundance data from HN‐CD tissues and reduced albumin level.

## Data Availability

The datasets generated or analysed during the current study are included in this article and in its Supplementary material files, which can be found with the online version of this article. Raw data on proteomics are submitted to Open Access database PRIDE–roteomics Identification Database (ebi.ac.uk). The population models for Simcyp are available on request from the corresponding authors, also uploaded to the user group website https://members.certara.co.uk/simcyp/customerrepository/Summary?id=110.
