# Supplementary material for: Variability in intestinal drug metabolizing enzymes and transporters in Crohn's disease and potential impact on oral drug absorption
Source: Br J Clin Pharmacol. 2025 Mar 4;91(7):2028–44. doi: 10.1002/bcp.70019 (PMC12199113; doi:10.1002/bcp.70019)
Supplement: Supplementary file 1 — Table S1. Demographic and clinical details of Crohn's disease (CD) patients. Table S2. Demographic details of healthy subjects. Table S3. High intensity unique peptide sequences assigned to each DMET and used to quantify their abundance in inflamed, histologically normal CD and healthy samples based on HiN label‐free methodology.3 Table S4. Input abundance values of drug‐metabolizing enzymes and transporters in the Simcyp Simulator for the created active CD population relative to healthy baseline based on data generated in the present study. Table S5. Specifics of the PBPK‐based simulation workflows for budesonide and midazolam implemented in the Simcyp Simulator and their corresponding trial design parameters. Table S6. Abundance (pmol/g of mucosal tissue) of CYP enzymes, UGT enzymes, non‐CYP/non‐UGT enzymes, ABC transporters, SLCs in inflamed Crohn's disease (I‐CD), histologically normal Crohn's disease (HN‐CD) and healthy ileum samples. Data are presented as mean, standard deviation of the mean (SD) and coefficient of variation (%CV). Table S7. Abundance (pmol/g of mucosal tissue) of CYP enzymes, UGT enzymes, non‐CYP/non‐UGT enzymes, ABC transporters, SLCs in inflamed Crohn's disease (I‐CD), histologically normal Crohn's disease (HN‐CD) and healthy colon samples. Data are presented as mean, standard deviation of the mean (SD) and coefficient of variation (%CV). Table S8. Comparison of predicted and observed 4 PK parameters and their fold change in active CD populations with the different applied models (M‐1, M‐2, M‐3 and M‐4) of oral budesonide‐controlled release formulation under fed conditions. Table S9. Comparison of predicted and observed5 PK parameters and their fold change in active CD populations with the different applied models (M‐1, M‐2, M‐3 and M‐4) of oral midazolam solution formulation under fasted conditions. Figure S1. Prediction of budesonide plasma concentration for healthy subjects after administration of (A) systemic 0.5 mg intravenous (IV) dos [file BCP-91-2028-s001.docx]

**Supplementary Material**

# **Variability in Intestinal Drug Metabolizing Enzymes and Transporters in Crohn’s Disease and the Potential Impact on Oral Drug Absorption**

*Sarah Alrubia, Brahim Achour, Zubida M. Al-Majdoub, Amin Rostami-Hodjegan, Jill Barber*

## **Supplementary Methods**

### **Materials**

Unless otherwise indicated, all chemicals were supplied by Sigma-Aldrich (Poole, UK). All solvents were HPLC grade and supplied by Thermo Fisher Scientific (Paisley, UK). Lysyl endopeptidase (Lys-C) was purchased from Wako (Osaka, Japan). Sequencing-grade modified trypsin was supplied by Promega (Southampton, UK). Complete Mini, EDTA-free protease inhibitor cocktail tablets were supplied by Roche (Mannheim, Germany). BCA protein concentration measuring kit was obtained from ThermoFisher Scientific (Hemel Hempstead, UK). Amicon Ultra 0.5 mL centrifugal filters at 10-kDa molecular weight cut-off (Merck Millipore, Nottingham, UK)

### **Intestine Samples and Donor Demographics**

The handling procedures for both sources have been aligned as much as possible to minimize variability. Both biobanks follow standardized protocols for sample collection and storage where the samples were fresh-frozen under -80°C. Additions of spiked-in BSA internal standard served as technical controls for protein quantification allowing normalization and correction for potential systematic biases.

### **Homogenate Preparation for Proteomics**

The extracted homogenates were stored in aliquots at -80°C until further processing. The protein content of ileum and colon homogenates was determined using a triplicate BCA assay with bovine serum albumin (BSA) as a standard. For proteomics, 70 μg homogenate protein from each sample was spiked with 0.126 μg BSA as internal standard, and the samples subjected to filter-aided sample preparation (FASP), as previously described.^1^

### **Liquid Chromatography-Tandem Mass Spectrometry (LC-MS/MS)**

LC mobile phase A (0.1% formic acid in water) and mobile phase B (0.1% formic acid in acetonitrile) were used to run a 90-min gradient. Peptides were resolved on Charged Surface Hybrid (CSH) C18 analytical column (75 mm x 250 μm inner diameter, 1.7 μm particle size) (Waters, UK). A 1 μl aliquot of each sample was transferred to a 5 μl loop and loaded onto the column at a flow rate of 300 nl/min for 5 min at 5% B. The loop was then taken out offline and the flow was reduced to 200 nl/min in 0.5 min. Peptides were separated using a gradient from 5% to 18% B in 63.5 min, then from 18% to 27% B in 8 min, and finally from 27% B to 60% B in 1 min. The column was washed with 60% B for 3 min before re-equilibration to 5% B in 1 min. At 85 min, the flow was increased to 300 nl/min until the end of the run. Peptides were selected for fragmentation automatically by data-dependant acquisition (DDA) with an MS scan window between m/z 300 and 1750. The top 12 peptides with a charge state of 2+ to 4+ were selected with dynamic exclusion set at 15 seconds. The MS resolution was set at 120,000 with an AGC target of 3E6 and a maximum fill time set at 20 ms. The MS2 resolution was set to 30,000, with an AGC target of 2E5, a maximum fill time of 45 ms, isolation window of 1.3 m/z and a collision energy of 28 eV.

### **Data Analysis and Protein Quantification**

To measure the abundance of identified CYPs, UGTs, non-CYP/non-UGT drug-metabolising enzymes, ABC transporters and SLCs, the average intensity of the three most abundant non-conflicting unique peptides was used to quantify the identified targeted proteins in relation to BSA at known abundance in each sample. When three unique peptides were not available in a sample, the average intensity of two unique peptides was used for quantification. A protein was considered quantifiable in intestine homogenate samples if it was identified by at least two unique peptides and it was detected in a sufficient number of samples [at least 2 samples/group/segment (except for HN-CD ileum as there were only 2 samples in this group; if the protein was not detected in this group but detected in an adequate number of samples in the other groups from the same segment, it was included)].

The abundance of each target protein was calculated using equation 1, as previously reported.^2^

$\left[ prtein \right]=\left[ BSA \right]\times\frac{(\sum_{i=1}^{n} I_{rank\left( i \right)}/n)}{(\sum_{j=1}^{n} I_{rank\left( j \right)}/m)}$ **(1)**

Where [Protein] is a target protein abundance and [BSA] is the abundance of the BSA internal standard, both measured in pmol/mg homogenate protein. The fractions reflect the ratio of the average intensity of the highest intensity unique peptides for each protein (n peptides) relative to the reference BSA (m peptides), where n and m = 2 or 3. Known concentration of BSA is used in all samples (26 pmol/mg of homogenate protein). The calculated ileum and colon mucosal abundances were then expressed in units of pmol/g of mucosal tissue by scaling up protein concentrations in homogenates (pmol/mg homogenate) using the amount of tissue prepared for homogenisation (mucosal weight in grams) for each sample.

**Table S1.** Demographic and clinical details of Crohn’s disease (CD) patients.

| **Sample ID** | **Tissues source** | **Gender** | **Age at surgery (year)** | **Ethnicity** | **Height (m)** | **Weight (kg)** | **BMI** | **Smoking** | **Drinking** | **Tissue classification** | **Medical history** | **Medication history** |
| --- | --- | --- | --- | --- | --- | --- | --- | --- | --- | --- | --- | --- |
| **328a** | Colon | Female | 38 | N/A | 1.74 | 53 | 17.51 | Yes (recent ex) | Yes occasionally | Diseased | Crohn’s disease | Methotrexate 4 years ago |
| **328b** |  |  |  |  |  |  |  |  |  | Histologically normal |  |  |
| **1942a** | Colon | Female | 25 | N/A | 1.57 | 42 | 17.04 |  | N/A | Diseased-Active CD with extensive ulceration | Crohn's diagnosed in 2003. Failure to all medications including adalimumab, infliximab, tacrolimus, vedolizumab, ustekinumab and anti-MAP therapy | Azathioprine |
| **1942b** |  |  |  |  |  |  |  |  |  | Histologically normal |  |  |
| **1940a** | Ileum | Female | 62 | Caucasian- British | 1.65 | 58.06 | 21.33 | No | N/A | Diseased- Patchy mild to moderate transmural chronic | Bowel resection (2013), bile salt malabsorption, reflux | Ustekinumab, Iron tablets, B12 injections, Cholestyramine, Omeprazole, Azathioprine, Fortisip |
| **1940b** |  |  |  |  |  |  |  |  |  | Histologically normal |  |  |
| **974** | Colon | Male | 18 | N/A | 1.8 | 55.2 | 17.04 | No | No | Diseased | Crohn's disease, recently treated for latent Tuberculosis | Laxido, Adalimumab, Azathioprine |
| **156** | Colon | Male | 39 | Caucasian-Irish | 1.8 | 111 | 34.26 | No | Yes 25 units per week | Diseased | Crohn’s disease, laprotomy (2005), reversal ileostomy (2005), gout wrist | Candesartan, Salbutamol, Loperamide, Buscupan, Mesalazine, Allupurinol |
| **1569** | Colon | Female | 31 | Caucasian-British | 1.65 | 74 | 27.18 | Ex - stopped 6yrs ago. E-cig currently | No | Diseased | Anorectal strictoplasty (2016), c-section x2 (2008 & 2009), drainage of fistula (2006), rectal abscess (2005), abdominal pain, heart murmur (as child), heartburn (reflux), low BP, tonsillectomy, vit. D deficiency | Omeprazole, Azathioprine |
| **1265b** | Colon | Male | 46 | Caucasian-British | 1.84 | 110 | 32.14 | Yes | N/A | Histologically normal | post-traumatic stress disorder, Crohn's disease with stricture formation (ileum) | Quetiapine, Mirtazapine, Co-codamol, Zopiclone |
| **1265a** | Terminal Ileum |  |  |  |  |  |  |  |  | Diseased |  |  |
| **2055** | Colon | Male | 30 | Pakistani | 1.87 | 120 | 34.32 | Cannabis for pain relief | N/A | Diseased | Anxiety, low mood | Loperamide, Mesalazine (octasa) |
| **917** | Ileum | Male | 23 | Caucasian-Irish | 1.87 | 84.6 | 24.19 | No | Occasionally | Diseased | Mild asthma, heartburn, ileal Crohn's disease | Prednisolone, Ciprofloxacin, Metronidazole, Omeprazole, Tramadol |
| **304** | Ileum | Female | 27 | Caucasian- British | N/A | 78 | N/A | Yes | No | Diseased | Terminal ileal Crohn's disease | Azathioprine, Movicol, Docusate sodium |
| **844b** | Colon | Female | 51 | N/A | 1.62 | 61 | 23.24 | N/A | N/A | Histologically normal | IBD, Ileal Crohn's disease | Seretide, Folic acid |
| **844a** | Ileum |  |  |  |  |  |  |  |  | Diseased |  |  |
| **1004a** | Ileum | Female | 19 | Caucasian- British | 1.62 | 46 | 17.53 | No | No | Diseased- mild CD | Ileal Crohn's disease | Azathioprine, Adalimumab |
| **1004b** |  |  |  |  |  |  |  |  |  | Histologically normal |  |  |
| **2003a** | Colon | Male | 68 | Caucasian-Irish | 1.8 | 91.2 | 28.14 | Ex | N/A | Diseased | Piles tied (2016), HTN, hypercholesterolaemia, heartburn, anaemia, diverticulitis, Crohn's | Amlodipine |
| **2003b** |  |  |  |  |  |  |  |  |  | Histologically normal |  |  |
| N/A, No available information; BP, blood pressure; IBD, inflammatory bowel disease; Ex, previous smoker; HTN, hypertension. | | | | | | | | | | | | |

**Table S2.** Demographic details of healthy subjects.

| **Sample ID** | **Tissues source** | **Gender** | **Age (year)** | **Ethnicity** | **Tissue classification** | **Cause of death** | **PMI (h)** |
| --- | --- | --- | --- | --- | --- | --- | --- |
| **F-28** | Colon, Descending | Female | 50 | Caucasian | Healthy | Car accident | 4 |
| **208A** | Colon, Transverse | Female | 78 | Caucasian | Healthy | Cardiovascular disease, unspecified | 5 |
| **S3-13** | Colon, Sigmoid | Male | 30 | Caucasian | Healthy | Car accident | 4 |
| **S4-12** | Colon, Descending | Male | 30 | Caucasian | Healthy | Car accident | 4 |
| **M-28** | Colon, Descending | Male | 54 | Caucasian | Healthy | Injuries in the abdomen | 4 |
| **1C-10** | Ileum | Male | 33 | Caucasian | Healthy | Traumatic injury | 4 |
| **S13-20** | Ileum | Male | 48 | Caucasian | Healthy | Mechanical trauma | 1 |
| **S3-26** | Ileum | Male | 30 | Caucasian | Healthy | Car accident | 4 |
| **208A** | Ileum | Female | 78 | Caucasian | Healthy | Cardiovascular disease, unspecified | 5 |
| **90-12-23A** | Ileum | Female | 65 | Caucasian | Healthy | Acute myocardial infarction | 9 |
| PMI, Postmortem interval. | | | | | | | |

**Table S3.** High intensity unique peptide sequences assigned to each DMET and used to quantify their abundance in inflamed, histologically normal CD and healthy samples based on HiN label-free methodology.^3^

| **Protein target** | **Peptide sequence** | **Peptide sequence** | **Peptide sequence** | **Subcellular fraction localisation** | **Detected in ileum/colon** |
| --- | --- | --- | --- | --- | --- |
| **CYP1A2** | IGSTPVLVLSR | YLPNPALQR |  | Endoplasmic reticulum membrane, microsomes | Both |
| **CYP20A1** | NHGTVWSEIGK | SNFALLLK | GFLDGSLDK*  LVVSLGTVDVLK** |  | Both |
| **CYP27A1** | VVLAPETGELK | EIEVDGFLFPK | DFAHMPLLK | Mitochondrion membrane | Both |
| **CYP51A1** | SPIEFLENAYEK | NEDLNAEDVYSR | TVCGENLPPLTYDQLK | Endoplasmic reticulum membrane, microsomes | Both |
| **CYP2C18** | YIDLLPTNLPHAVTCDVK | VQEEIECVVGR |  |  | Ileum |
| **CYP2C19** | NLAFMESDILEK | GHFPLAER |  |  | Ileum |
| **CYP2D6** | AFLTQLDELLTEHR | VQQEIDDVIGQVR |  |  | Ileum |
| **CYP2S1** | MAQEEQNPGTEFTNK | DLVDAFLLK | ELGAGQAPSLGDR*  HEAFLPFSLGK** |  | Both |
| **CYP3A4** | GFCMFDMECHK | LSLGGLLQPEK | VWGFYDGQQPVLAITDPDMIK |  | Ileum |
| **CYP3A5** | SLGPVGFMK | SAISLAEDEEWK |  |  | Ileum |
| **CYP4F2** | NWFWGHQGMVNPTEEGMR | HVTQDIVLPDGR |  |  | Both |
| **CYP4F11** | TLTQLVTTYPQGFK | DMIFYGFLK |  |  | Ileum |
| **CYP4F12** | TLPTQGIDDFFK | SITNASAAIAPK |  |  | Both |
| **UGT1A1** | DSAMLLSGCSHLLHNK | DGAFYTLK |  | Endoplasmic reticulum membrane | Ileum |
| **UGT1A10** | TYSTSYTLEDQNR | GHEVVVVMPEVSWQLER |  |  | Both |
| **UGT2A3** | LNDFFVEIR | VILEELIVR* LYDWIPQNDLLGHPK** | TMTSEDLLR*  VLVWPCDMSHWLNVK** |  | Both |
| **UGT2B7** | TELENFIMQQIK | ADVWLIR |  |  | Both |
| **UGT2B17** | SVINDPIYK | NDLEDFFMK | LCEDAVLNK*  WTYSISK** |  | Both |
| **ALPI** | QVPDSAATATAYLCGVK | QAAEALDAAK | GFYLFVEGGR | Plasma membrane | Ileum |
| **AOX1** | GLHGPLTLNSPLTPEK | LILNEVSLLGSAPGGK | VFFGEGDGIIR | Cytoplasm and Cytosol | Both |
| **CES1** | FLSLDLQGDPR | EGYLQIGANTQAAQK | TTTSAVMVHCLR | Endoplasmic reticulum | Both |
| **CES2** | ADHGDELPFVFR | EEILAINK | TTHTGQVLGSLVHVK |  | Both |
| **EPHX1** | FSTWTNTEFR | ENLGQGWMTQK | IIPLLTDPK |  | Both |
| **EPHX2** | ILIPALMVTAEK | GLLNDAFQK | VCEAGGLFVNSPEEPSLSR |  | Both |
| **FMO3** | LVGPGQWPGAR | NNLPTAISDWLYVK |  |  | Both |
| **FMO5** | ALSQHPTLNDDLPNR | IAVIGGGVSGLSSIK* | WATQVFK** |  | Both |
| **MAOA** | IFFAGTETATK | IFSVTNGGQER | EIPTDAPWEAQHADK*  DIWVQEPESK** | Mitochondrion membrane | Both |
| **MAOB** | APLAEEWDNMTMK | LLHDSGLNVVVLEAR | YVDLGGSYVGPTQNR |  | Both |
| **MGST1** | VFANPEDCVAFGK | IYHTIAYLTPLPQPNR | MMLMSTATAFYR | Endoplasmic reticulum | Both |
| **MGST2** | VTPPAVTGSPEFER | HLYFWGYSEAAK |  |  | Both |
| **MGST3** | IASGLGLAWIVGR | VEYPIMYSTDPENGHIFNCIQR | VLYAYGYYTGEPSK |  | Both |
| **NAT1** | NYIVDAGFGR | LDLETLTDILQHQIR | SYQMWQPLELISGK** | Cytoplasm and Cytosol | Both |
| **NAT2** | DNTDLVEFK | TLTEEEVEEVLK |  |  | Ileum |
| **SULT1A1** | VHPEPGTWDSFLEK | APGIPSGMETLK |  |  | Both |
| **SULT1A2** | VYPHPGTWESFLEK | VPGIPSGMETLK* | THLPLALLPQTLLDQK** |  | Both |
| **SULT1B1** | THLPTDLLPK | IIHHTSFEVMK*  MIYLAR** | TSGIEQLEK*  NLNDEILDR** |  | Both |
| **SULT1E1** | NHFTVALNEK | LIHFLER |  |  | Both |
| **SULT2A1** | DEDVIILTYPK | TLEPEELNLILK |  |  | Both |
| **TPMT** | NQVLTLEEWQDK | SWGIDCLFEK |  |  | Both |
| **TXN** | PFFHSLSEK | CMPTFQFFK | TAFQEALDAAGDK | Nucleus and Cytoplasm | Both |
| **ABCB1**  **(P-gp, MDR1)** | FYDPLAGK | IATEAIENFR | AGAVAEEVLAAIR | Plasma membrane | Both |
| **ABCB3 (TAP2)** | EAVGGLQTVR | AHQILVLQEGK*  SFGAEEHEVCR** | EIQDAVAR*  QDLGFFQETK** | Endoplasmic reticulum | Both |
| **ABCB7** | VLSGISFEVPAGK | LAGLHDAILR | VAISLGFLGGAK*  AMLSYVWPK** | Mitochondrion membrane | Both |
| **ABCB8** | IVALVGQSGGGK | AMGVADEALGNVR |  |  | Both |
| **ABCB10** | VYLMQTSGQR | VAEVANAVAFIR |  |  | Ileum |
| **ABCB11 (BSEP)** | AADTIIGFEHGTAVER | STALQLIQR |  | Plasma membrane | Both |
| **ABCC1 (MRP1)** | DGAFAEFLR | SPVYSHFNETLLGVSVIR |  |  | Colon |
| **ABCC2 (MRP2)** | YLGGDDLDTSAIR | LTIIPQDPILFSGSLR |  |  | Both |
| **ABCC3 (MRP3)** | SPQSFFDTTPSGR | IDGLNVADIGLHDLR | SSMTLCLFR* SQLTIIPQDPILFSGTLR** |  | Both |
| **ABCC4 (MRP4)** | APVLFFDR | AEAAALTETAK |  |  | Both |
| **ABCC6 (MRP6)** | SSLASGLLR | APETEPFLR |  |  | Both |
| **ABCD1** | DQVIYPDSVEDMQR | VHEMFQVFEDVQR |  | Peroxisome membrane | Ileum |
| **ABCD3** | EYLDNVQLGHILER | IANPDQLLTQDVEK | ITELMQVLK |  | Both |
| **ABCE1** | CPFGALSIVNLPSNLEK | TQAIVCQQLDLTHLK | NTVANSPQTLLAGMNK** | Mitochondrion and Cytoplasm | Both |
| **ABCG2 (BCRP)** | ENLQFSAALR | SSLLDVLAAR | VIQELGLDK | Plasma membrane | Both |
| **SLC15A1 (PEPT1)** | CGFNFTSLK | HTLLVWAPNHYQVVK | WTLQATTMSGK | Membrane protein | Ileum |
| **SLC16A1 (MCT1)** | SITVFFK | DLHDANTDLIGR |  | Plasma membrane | Both |
| **SLC51A**  **(OST-α)** | VGYETFSSPDLDLNLK | YTADLLEVLK |  | Plasma membrane | Both |
| **SLC51B**  **(OST-β)** | ETPEVLHLDEAK | DHNSLNNLR |  |  | Both |
| **SLCO1A2 (OATP1A2)** | IYDSTTFR | EGLETNADIIK |  | Plasma membrane | Both |
| **SLCO1B1 (OATP1B1)** | LNTVGIAK | YVEQQYGQPSSK |  |  | Ileum |
| **SLCO2B1 (OATP2B1)** | VLLQTLR | SSPAVEQQLLVSGPGK |  |  | Both |
| *only detected adequately in ileum samples; **only detected adequately in colon samples | | | | | |

**Table S4.** Input abundance values of drug-metabolising enzymes and transporters in the Simcyp Simulator for the created active CD population relative to healthy baseline based on data generated in the present study.

| **Metabolising enzyme/ transporter** | **Small intestine (SI) (nmol/SI)** | | | | **Colon (nmol/colon)** | | | |
| --- | --- | --- | --- | --- | --- | --- | --- | --- |
|  | **Inflamed CD (n=6)** | **CV (%)** | **Histologically normal CD (n=2)** | **CV (%)** | **Inflamed CD (n=7)** | **CV (%)** | **Histologically normal CD**  **(n=5)** | **CV (%)** |
| CYP3A4 | 8.6 | 75.3 | 15.6 | 81.4 | Change was assumed to be similar to the change detected in ileum as it was below LLOQ (1.88 pmol/g of mucosal tissue) in our colon tissues | | | |
| CYP3A5 | 2.48 | 100 | 0.18 | N/A |  |  |  |  |
| CYP2D6 | 0.39 | 67.5 | N/A | N/A |  |  |  |  |
| UGT2B7 | 1.28 | 96.6 | 1.6 | 65.1 | N/A | | | |
| P-gp* | 0.11 | 88.2 | 0.08 | 61.7 | 0.3 | 73.6 | 0.19 | 89.1 |
| MRP2* | 0.19 | 43.7 | 0.35 | N/A | No change from default healthy | | | |
| BCRP* | 0.17 | 58.2 | 0.1 | 11.3 | No change from default healthy | | 0.22 | 48 |
| OATP2B1* | 0.15 | 79.9 | 0.15 | N/A | No change from default healthy | | | |
| N/A, not applicable as no data as the value could not be calculated due to insufficient data points or lack of detection, thus default healthy values were used. *Ileum and colon transporter distribution is incorporated and expressed relative to the abundance in the jejunum. | | | | | | | | |
| Note: Whenever a protein was not detected in colon but detected in ileum and its abundance level was available in Simcyp, then the assumption of its reduction was made based on the reduction detected in ileum tissues from the same tissue nature (I-CD or HN-CD). Also, if a protein was not detected or its expression change was not ≥2 fold from healthy in either ileum or colon, then the original abundance in Simcyp was used without change. | | | | | | | | |

**Table S5.** Specifics of the PBPK-based simulation workflows for budesonide and midazolam implemented in the Simcyp Simulator and their corresponding trial design parameters.

| **Trial parameter** | **Budesonide** ^4^ | **Midazolam** ^5^ |
| --- | --- | --- |
| Oral Dose | 18 mg | 0.1 mg |
| Number of individuals / trials | 10 trials with 10 virtual individuals (100 virtual subjects) | |
| CD population age range | 21–63 years | 25–65 years |
| CD female / male | 50% female (3/3) 3 active and 3 inactive | 87% (7/1) all active |
| Duration of the study (hr) | 40 | 10 |
| Fed / fasted | Fed | Fasted |

## **Supplementary Results**

**Table S6.** Abundance (pmol/g of mucosal tissue) of CYP enzymes, UGT enzymes, non-CYP/non-UGT enzymes, ABC transporters, SLCs in inflamed Crohn’s disease (I-CD), histologically normal Crohn’s disease (HN-CD) and healthy ileum samples. Data are presented as mean, standard deviation of the mean (SD) and coefficient of variation (%CV).

| **Ileum protein target abundance (pmol/g of mucosal tissue)** | **Healthy (n=5)** | | **Inflamed Crohn’s disease (I-CD, n=6)** | | **Histologically normal Crohn’s disease (HN-CD, n=2)** |
| --- | --- | --- | --- | --- | --- |
|  | **Mean±SD** | **CV (%)** | **Mean±SD** | **CV (%)** | **Mean±SD** |
| **CYP1A2** | 2±1.5 | 73.7 | 0.9±0.5 | 53.3 | 1.1±1.4 |
| **CYP20A1** | 4.5±2.1 | 46.7 | 2.1±0.7 | 35.3 | 2.2±1.6 |
| **CYP27A1** | 38±14.8 | 39 | 15.5±13.4 | 86 | 13.7±13.3 |
| **CYP51A1** | 6.3±2.7 | 41.9 | 2.4±2.3 | 98.2 | 1±0.6 |
| **CYP2C18** | 3.9±3.2 | 82 | 0.8±0.7 | 84.3 | 2±1.9 |
| **CYP2C19** | 2.2±3.1 | 140 | 0.7±0.2 | 32.3 | 0.8±0.5 |
| **CYP2D6** | 6.3±5.5 | 87.2 | 2.2±1.5 | 67.5 | N/A |
| **CYP2S1** | 12.9±6.7 | 51.7 | 5.6±2.5 | 44 | 5.6±6.9 |
| **CYP3A4** | 50.8±38.4 | As 75.6 | 6.7±5 | 75.3 | 12.1±9.9 |
| **CYP3A5** | 18±12.7 | 70.5 | 1.9±2.1 | 111.6 | 0.1±N/A |
| **CYP4F2** | 25.6±26.1 | 102 | 3.5±2.9 | 81.2 | 1.7±1.5 |
| **CYP4F11** | 5.5±4 | 72.8 | 1.1±0.4 | 39.1 | 5.9±N/A |
| **CYP4F12** | 9.9±12.4 | 125.6 | 2±1.8 | 89.3 | 1.6±0.9 |
| **UGT1A1** | 32.5±34.4 | 105.7 | 10.5±5.5 | 51.9 | 0.4±0.4 |
| **UGT1A10** | 9±10.2 | 113.7 | 2.3±1.9 | 82.4 | 2.7±N/A |
| **UGT2A3** | 56.5±25.5 | 45.1 | 14.1±13.4 | 95.2 | 23.3±23.4 |
| **UGT2B7** | 13.6±11.4 | 84.2 | 3.8±3.7 | 96.6 | 4.7±3.1 |
| **UGT2B17** | 309±239.4 | 77.5 | 222.6±160.7 | 72.2 | 201.1±220.3 |
| **ALPI** | 26.8±25.7 | 96 | 6.4±4.4 | 68.7 | 15.9±9 |
| **AOX1** | 6.2±2.9 | 46 | 1.5±0.8 | 50.9 | 1.4±1.7 |
| **CES1** | 3.1±2.2 | 71.7 | 1.6±1.4 | 90.6 | 2±2 |
| **CES2** | 67.4±51.3 | 76 | 56.6±65.7 | 116.1 | 59.3±72.7 |
| **EPHX1** | 37.1±17.4 | 46.8 | 10.8±7.1 | 65.5 | 18.8±4.5 |
| **EPHX2** | 7.5±3.1 | 41.4 | 7±6.8 | 97.5 | 3.3±2.5 |
| **FMO3** | 1.5±1.2 | 76.5 | 0.6±0.6 | 99.7 | 0.4±0.4 |
| **FMO5** | 5.4±2.8 | 51.4 | 2.2 ±1.7 | 76.8 | 2.6±3.5 |
| **MAOA** | 482.9±153.4 | 31.8 | 94.5±63.3 | 67 | 128.3±100.4 |
| **MAOB** | 156.1±97.3 | 62.3 | 26.4±23.8 | 90.1 | 64±10.2 |
| **MGST1** | 49.4±32.3 | 65.4 | 26.4±17.3 | 65.4 | 12±16.4 |
| **MGST2** | 25.9±10.2 | 39.4 | 10.5±6.4 | 61.5 | 13.1±10.3 |
| **MGST3** | 60.1±47.3 | 78.7 | 17.4±17.6 | 101.5 | 9.6±13 |
| **NAT1** | 7.1±4.3 | 60.5 | 0.9±0.6 | 63.2 | 0.8±1 |
| **NAT2** | 2.1±1.6 | 79.2 | 0.6±0.1 | 23.7 | N/A |
| **SULT1A1** | 45.5±23.6 | 51.8 | 5.9±8.7 | 145.9 | 3.1±N/A |
| **SULT1A2** | 156±75.5 | 48.4 | 10.6±13.8 | 130.5 | 9.7±1 |
| **SULT1B1** | 59.4±45.2 | 76.1 | 9.3±10.1 | 108 | 5.1±6 |
| **SULT1E1** | 10.9±7.4 | 67.6 | 0.5±0.5 | 92.8 | 0.3±0.3 |
| **SULT2A1** | 17.9±16.4 | 91.5 | 0.6±0.4 | 63 | 0.9±0.3 |
| **TPMT** | 2.5±1.5 | 59.2 | 1.1±1.1 | 99.9 | 0.6±0.6 |
| **TXN** | 50.6±21.1 | 41.7 | 36.1±28.4 | 78.6 | 14.8±12.2 |
| **ABCB1 (P-gp)** | 20.3±16 | 78.7 | 6±5.3 | 88.2 | 3.8±2.4 |
| **ABCB3 (TAP2)** | 13.9±5.5 | 39.6 | 9.3±6.1 | 65.6 | 1.3±N/A |
| **ABCB7** | 5±2.4 | 49.1 | 1.4±0.8 | 55.4 | 0.7±0.2 |
| **ABCB8** | 3.6±0.5 | 14.9 | 0.9±0.7 | 73.5 | 0.3±0.1 |
| **ABCB10** | 1.5±1.4 | 91.3 | 1±1.2 | 118.5 | 1.1±0.4 |
| **ABCB11 (BSEP)** | 4.3±3.2 | 75 | 1.2±0.7 | 60.6 | 0.8±0.9 |
| **ABCC2 (MRP2)** | 1.7±1.8 | 103.8 | 0.4±0.2 | 43.7 | 0.7±N/A |
| **ABCC3 (MRP3)** | 4±2.6 | 65.6 | 4.5±1.3 | 28.4 | 1.5±2.1 |
| **ABCC4 (MRP4)** | 0.8±0.7 | 87.3 | 0.3±0.2 | 65.3 | 0.3±0.3 |
| **ABCC6 (MRP6)** | 3±2.2 | 74.4 | 1±0.6 | 61.9 | 5.5±7.6 |
| **ABCD1** | 6.2±6.6 | 106.8 | 0.7±0.2 | 36.7 | 0.2±N/A |
| **ABCD3** | 30.6±17 | 55.5 | 15.3±5 | 32.6 | 11.7±15.4 |
| **ABCE1** | 5.7±4.8 | 85.3 | 0.9±0.7 | 74.7 | 0.2±N/A |
| **ABCG2 (BCRP)** | 6.3±4.9 | 77.9 | 3.1±1.8 | 58.2 | 1.9±0.2 |
| **SLC15A1 (PEPT1)** | 9.6±7.7 | 80.3 | 4.6±4.7 | 102.9 | 0.8±0.1 |
| **SLC16A1 (MCT1)** | 1.4±0.9 | 61.6 | 0.8±0.4 | 54.9 | 0.6±0.04 |
| **SLC51A (OST-α)** | 7.8±4.2 | 53.7 | 3.8±2.2 | 59.3 | 4.1±4.9 |
| **SLC51B (OST-β)** | 1±0.5 | 52.7 | 0.9±0.4 | 47.4 | 1.1±N/A |
| **SLCO1A2 (OATP1A2)** | 1.1±0.8 | 74.7 | 0.4±0.3 | 72.4 | 0.7±N/A |
| **SLCO1B1 (OATP1B1)** | 0.3±0.1 | 18.7 | 0.1±0.03 | 28.3 | 0.1±N/A |
| **SLCO2B1 (OATP2B1)** | 3±1.7 | 57.5 | 1.1±0.9 | 79.9 | 1.1±N/A |
| N/A, no available data as the value could not be calculated due to insufficient data points (reported in only one sample in the group) or the target was not detected | | | | | |

**Table S7.** Abundance (pmol/g of mucosal tissue) of CYP enzymes, UGT enzymes, non-CYP/non-UGT enzymes, ABC transporters, SLCs in inflamed Crohn’s disease (I-CD), histologically normal Crohn’s disease (HN-CD) and healthy colon samples. Data are presented as mean, standard deviation of the mean (SD) and coefficient of variation (%CV).

| Colon protein target abundance (pmol/g of mucosal tissue) | Healthy (n=5) | | Inflamed Crohn’s disease (I-CD, n=7) | | Histologically normal Crohn’s disease (HN-CD, n=5) | |
| --- | --- | --- | --- | --- | --- | --- |
|  | **Mean±SD** | **%CV** | **Mean±SD** | **%CV** | **Mean±SD** | **%CV** |
| CYP1A2 | 1.2±0.6 | 50.7 | 0.6±0.5 | 81.4 | 0.7±0.7 | 102.3 |
| CYP20A1 | 2.7±1.7 | 61.6 | 3.1±1.7 | 56.8 | 2±1.3 | 63 |
| CYP27A1 | 7.8±5.8 | 74.6 | 6.9±5.6 | 80.9 | 1.3±0.9 | 65.6 |
| CYP51A1 | 4.3±1.4 | 33.3 | 4.9±3.7 | 75.1 | 3.1±3.8 | 122.5 |
| CYP2S1 | 8.5±3.9 | 45.3 | 4.7±4.2 | 90.5 | 2.7±1.6 | 59.9 |
| CYP4F2 | 2.3±1.9 | 85.7 | 0.7±0.4 | 63.2 | 0.7±0.2 | 30.4 |
| CYP4F12 | 7±4.9 | 69.6 | 1.8 ±1.1 | 58.7 | 5±6.3 | 127.1 |
| UGT1A10 | 6.7±5.9 | 88.4 | 1.6±1.5 | 94.1 | 2.3±1.5 | 67.8 |
| UGT2A3 | 21±11.5 | 53.3 | 9.8±5.6 | 57 | 8.9±8.8 | 98.8 |
| UGT2B7 | 1.2±1.4 | 117.6 | 2±2.1 | 105.8 | 0.2±0.2 | 64.3 |
| UGT2B17 | 435.9±302 | 69.3 | 166.7±143.6 | 86.1 | 261.4±190.2 | 72.7 |
| AOX1 | 4.6±1.7 | 37.2 | 4±4.2 | 106.1 | 2.6±2.5 | 97.1 |
| CES1 | 4.8±2.5 | 51.1 | 6.8±8.7 | 128.2 | 5.4±5.2 | 96.2 |
| CES2 | 37±35.8 | 96.7 | 20.6±13.3 | 64.3 | 25.7±25.6 | 99.7 |
| EPHX1 | 33.9±6 | 17.8 | 19.2±14.5 | 75.7 | 19.6±16.2 | 82.3 |
| EPHX2 | 9.9±7.4 | 74.8 | 4.8±3.6 | 75.1 | 4.9±4.4 | 89.4 |
| FMO3 | 1±0.5 | 46.9 | 0.9±0.9 | 103.5 | 0.7±0.6 | 87.5 |
| FMO5 | 3.8±61.4 | 61.4 | 2.2±1.6 | 74.8 | 2.1±2.1 | 100.4 |
| MAOA | 217.2±131.5 | 60.5 | 83±72.4 | 87.2 | 84.3±59 | 70 |
| MAOB | 44.7±29.8 | 66.6 | 20.5±10.4 | 50.6 | 17.5±9.3 | 53.3 |
| MGST1 | 65±32.4 | 49.8 | 51.7±43.7 | 84.5 | 32.8±18.2 | 55.5 |
| MGST2 | 16.3±9.8 | 59.9 | 12±9.1 | 75.6 | 8.9±6.1 | 69.2 |
| MGST3 | 51.3±32.5 | 63.3 | 17.4±17.6 | 100.8 | 16±11 | 68.6 |
| NAT1 | 5.6±2.7 | 48.8 | 4.9±5.6 | 115.1 | 1.2±0.9 | 76 |
| SULT1A1 | 6.4±1.6 | 24.9 | 2.4±1.6 | 67.7 | 2±1.9 | 94.1 |
| SULT1A2 | 33.8±10.9 | 32.2 | 9.6±8 | 83.9 | 2.6±2.5 | 97.9 |
| SULT1B1 | 28.9±18.2 | 62.7 | 4.9±5.1 | 103.1 | 1.8±1.2 | 64.3 |
| SULT1E1 | 0.4±0.1 | 18.3 | 0.3±0.2 | 71 | 0.2±0.2 | 116.8 |
| SULT2A1 | 1±0.6 | 60.6 | 0.9±1.4 | 156.5 | 0.4±0.3 | 78 |
| TPMT | 1.6±1.1 | 69.1 | 0.8±0.6 | 72.3 | 0.7±0.4 | 56.9 |
| TXN | 101.8±76.4 | 75.1 | 60.4±42.8 | 70.9 | 52±39.9 | 76.7 |
| ABCB1 (P-gp) | 3.9±2.9 | 72.2 | 2.1±1.5 | 73.6 | 1.3±1.1 | 86.1 |
| ABCB3 (TAP2) | 7±3.5 | 50.3 | 12.9±11.5 | 89.5 | 3.4±5.8 | 169.3 |
| ABCB7 | 4.5±2.3 | 52.3 | 1.5±0.8 | 53 | 1.5±1.2 | 79.4 |
| ABCB8 | 2.2±0.8 | 35.4 | 0.2±0.2 | 77.9 | 0.4±0.6 | 136.8 |
| ABCB11 (BESP) | 5.2±3.1 | 59.1 | 2±2.5 | 125.2 | 1.4±0.8 | 57.8 |
| ABCC1 (MRP1) | 4.6±0.5 | 11.1 | 0.9±0.3 | 38.7 | 0.2±0.1 | 40.8 |
| ABCC2 (MRP2) | 0.5±0.3 | 56.1 | 0.9±0.8 | 86.2 | 0.8±0.8 | 103.3 |
| ABCC3 (MRP3) | 16.1±9.5 | 59.2 | 5.7±6 | 105.1 | 4.1±2.8 | 67.7 |
| ABCC4 (MRP4) | 0.7±0.2 | 23.3 | 0.8±0.6 | 76.6 | 0.4±0.3 | 66 |
| ABCC6 (MRP6) | 1±0.8 | 76.8 | 1.2±1.3 | 105.7 | 1.6±2.3 | 144.1 |
| ABCD3 | 20.3±11.7 | 57.9 | 9.6±8.2 | 85.2 | 8.8±5.6 | 63.9 |
| ABCE1 | 2.8±2.5 | 87.1 | 1.9±2.3 | 121.4 | 0.3±0.3 | 93.4 |
| ABCG2 (BCRP) | 2.5±0.7 | 27.7 | 2.1±1.9 | 91.8 | 0.9±0.5 | 48 |
| SLC16A1 (MCT1) | 6.1±3.8 | 62.4 | 0.9±0.5 | 53.6 | 3.1±4 | 127.1 |
| SLC51A (OST-α) | 2.3±1.2 | 50.8 | 1.2±0.6 | 52.1 | 1.9±2.4 | 129.9 |
| SLC51B (OST-β) | 1.1±0.7 | 62.8 | 0.4±0.5 | 135.4 | 0.6±0.7 | 110.1 |
| SLCO1A2 (OATP1A2) | 0.9±0.3 | 35.9 | 0.6±0.6 | 98.1 | 0.3±0.3 | 76.1 |
| SLCO2B1 (OATP2B1) | 1.4±0.6 | 45.7 | 1.4±1.8 | 130.7 | 1±1.2 | 118.1 |

**Table S8.** Comparison of predicted and observed ^4^ PK parameters and their fold change in active CD populations with the different applied models (M-1, M-2, M-3 & M-4) of oral budesonide-controlled release formulation under fed conditions.

| **Parameter** | **AUC_0-∞_ (nM*h)** | | | **Cmax (nM)** | | | **Tmax (h)** | | | **F (%)** | | |
| --- | --- | --- | --- | --- | --- | --- | --- | --- | --- | --- | --- | --- |
| **Model** | Predicted | Observed  Mean,  95% CI | Predicted/  Observed | Predicted | Observed  Mean,  95% CI | Predicted/  Observed | Predicted | Observed  Mean,  95% CI | Predicted/  Observed | Predicted | Observed  Mean,  95% CI | Predicted/  Observed |
| **M-1** | 105.4 | 114, (81.4-159.5) | 0.92 | 14.91 | 14.3,  (6-13.7) | 1.04 | 4.61 | 6, (3-8) | 0.77 | 29 | 20.5, (8.8-15) | 1.4 |
| **M-2** | 62.05 |  | 0.54 | 8.23 |  | 0.58 | 4.67 |  | 0.8 | 21 |  | 1.02 |
| **M-3** | 105.3 |  | 0.92 | 14.91 |  | 1.04 | 4.6 |  | 0.77 | 28 |  | 1.37 |
| **M-4** | 62 |  | 0.54 | 8.24 |  | 0.58 | 4.67 |  | 0.8 | 20 |  | 0.98 |
| M-1, Model 1 CD population with intestine DMET abundance data from I-CD tissues and normal albumin level; M-2, Model 2 CD population with intestine DMET abundance data from I-CD tissues and reduced albumin level; M-3, Model 3 CD population with intestine DMET abundance data from HN-CD tissues and normal albumin level; M-4, Model 4 CD population with intestine DMET abundance data from HN-CD tissues and reduced albumin level. 95% CI, 95% confidence interval. | | | | | | | | | | | | |

**Table S9.** Comparison of predicted and observed^5^ PK parameters and their fold change in active CD populations with the different applied models (M-1, M-2, M-3 & M-4) of oral midazolam solution formulation under fasted conditions.

| **Parameter** | **AUC0-∞ (nM*h)** | | | **Cmax (nM)** | | | **Tmax (h)** | | | **F (%)** | | |
| --- | --- | --- | --- | --- | --- | --- | --- | --- | --- | --- | --- | --- |
| **Model** | Predicted | Observed  Mean ±SD | Predicted/  Observed | Predicted | Observed  Mean ±SD | Predicted/  Observed | Predicted | Observed  Mean ±SD | Predicted/  Observed | Predicted | Observed  Mean ±SD | Predicted/  Observed |
| **M-1** | 8.6 | 14±6.38 | 0.61 | 3.7 | 8.4±5.13 | 0.44 | 0.53 | 0.53± 1.3 | 1 | 55 | 31±22 | 1.8 |
| **M-2** | 4.14 |  | 0.3 | 2.41 |  | 0.3 | 0.42 |  | 0.8 | 43 |  | 1.4 |
| **M-3** | 7.8 |  | 0.56 | 3.4 |  | 0.4 | 0.51 |  | 0.96 | 50 |  | 1.6 |
| **M-4** | 3.75 |  | 0.27 | 2.21 |  | 0.26 | 0.41 |  | 0.77 | 39 |  | 1.26 |
| M-1, Model 1 CD population with intestine DMET abundance data from I-CD tissues and normal albumin level; M-2, Model 2 CD population with intestine DMET abundance data from I-CD tissues and reduced albumin level; M-3, Model 3 CD population with intestine DMET abundance data from HN-CD tissues and normal albumin level; M-4, Model 4 CD population with intestine DMET abundance data from HN-CD tissues and reduced albumin level. | | | | | | | | | | | | |

***Figure S1:*** Prediction of budesonide plasma concentration for healthy subjects after administration of **A)** systemic 0.5 mg intravenous (IV) dose (n=24) with observed values from *(Thorsson et al., 1994)*^7^ and **B)** 18 mg oral (PO) solution (n=8) and **C)** 18 mg oral (PO) solution log scale with observed values from *(Edsbäcker et al., 2003)*^4^ in the fed state.


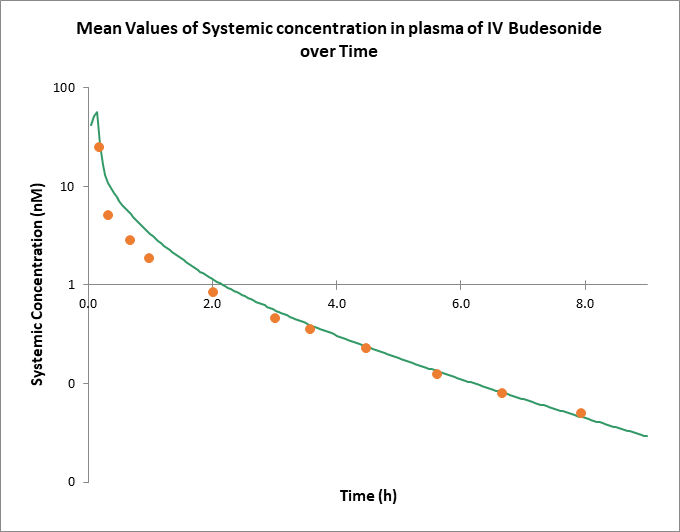


**A)**


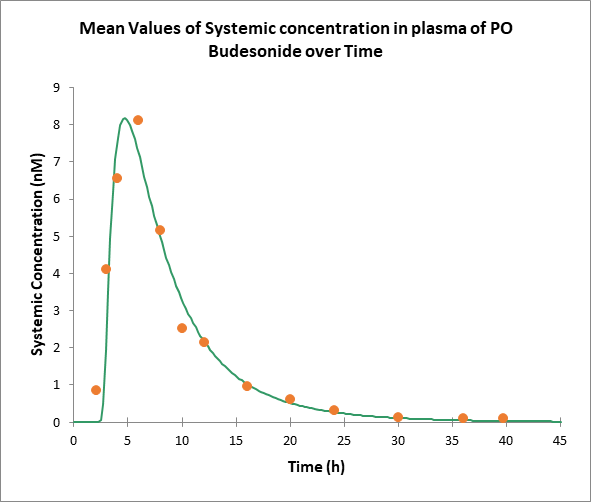


**B)**


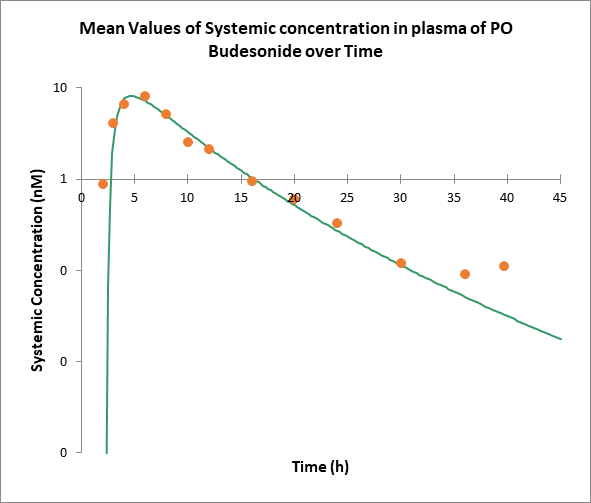


**C)**

***Figure S2:*** Prediction of Midazolam plasma concentration for healthy subjects (n=16) from *(Hohmann et al., 2015)*^8^ after administration of **A)** systemic 0.001 mg intravenous (IV) dose with observed values and **B)** 0.003 mg oral (PO) solution and **C)** 0.003 mg oral (PO) solution log scale with observed values in the fasted state.


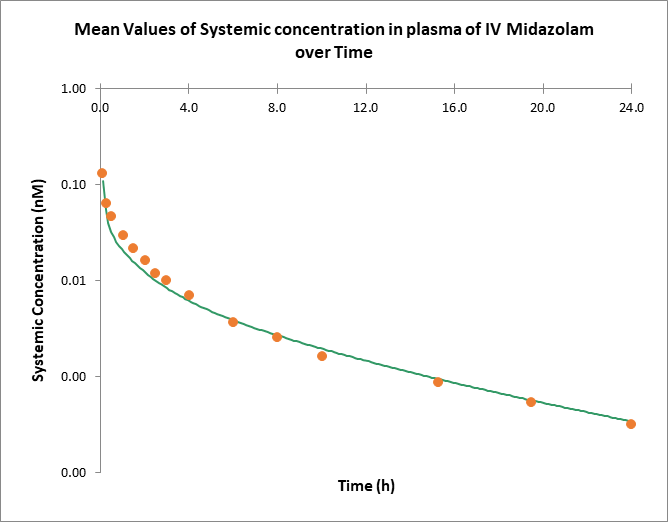


**A)**


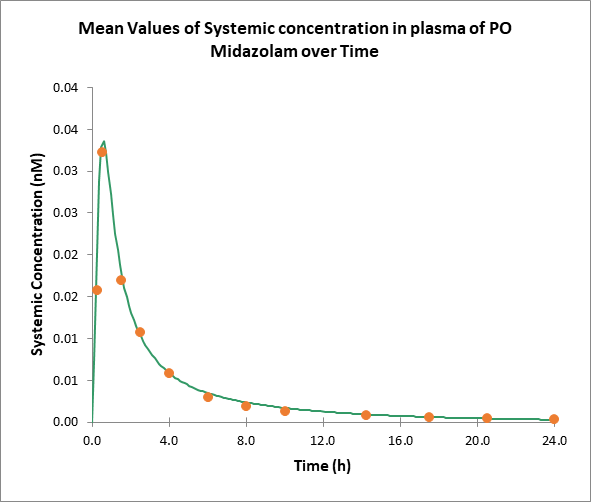


**B)**


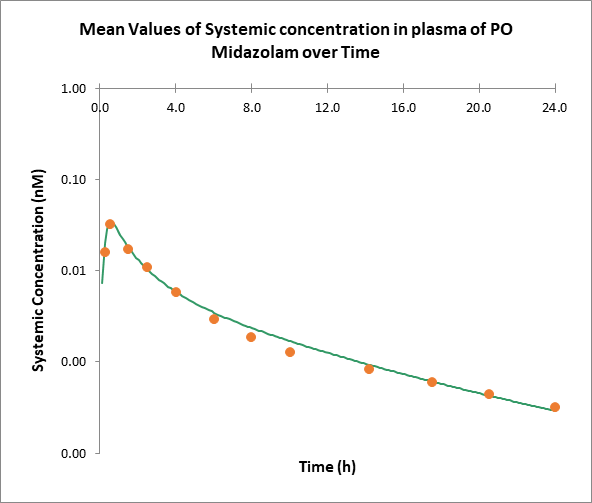


**C)**


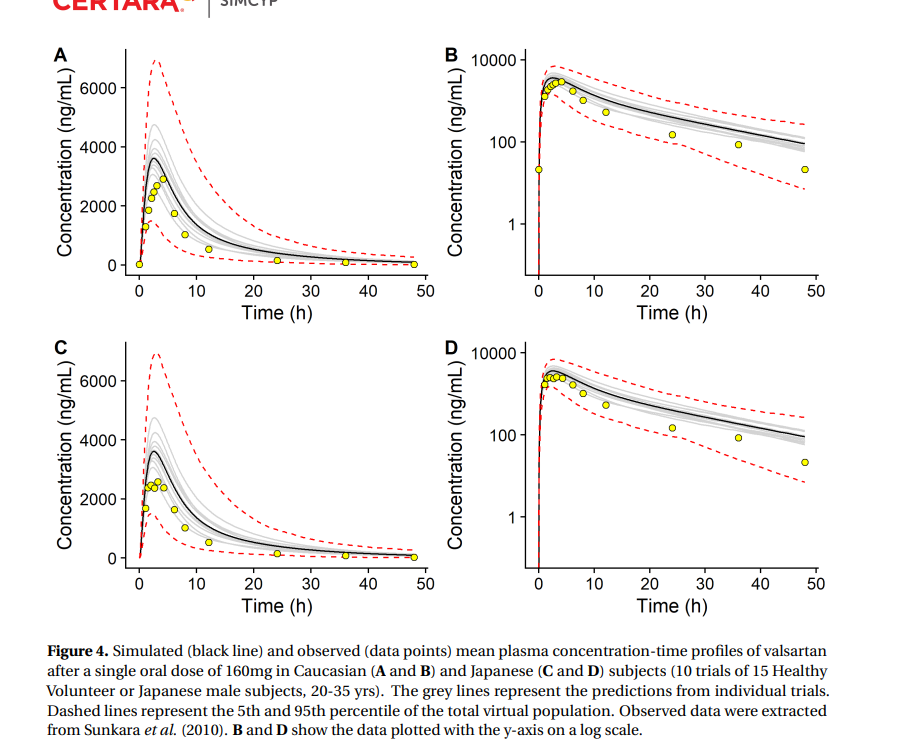


***Figure S3.*** Performance verification of valsartan drug profile in Simcyp simulator provide by Simcyp, Certara. Simulated (black line) and observed (data points) mean plasma concentration-time profiles of Valsartan after a single oral dose of 160 mg in Caucasian (A and B) and Japanese (C and D) subjects (10 trials of 15 healthy volunteers or Japanese male subjects, 20-35 yrs). The grey lines represent the prediction from individual trials. Dashed lines represent the 5^th^ and 95^th^ percentile of the total virtual population. Observed data were extracted from *(Sunkara et al., 2010)^6^.*B and D show the data plotted with the y-axis on a log scale.

***Figure S4.*** Technical variability in (A) ileum and (B) Colon DMETs presented as coefficients of variation (CV%) for different targets in a set of 3 ileum and 3 colon samples (prepared in triplicates).


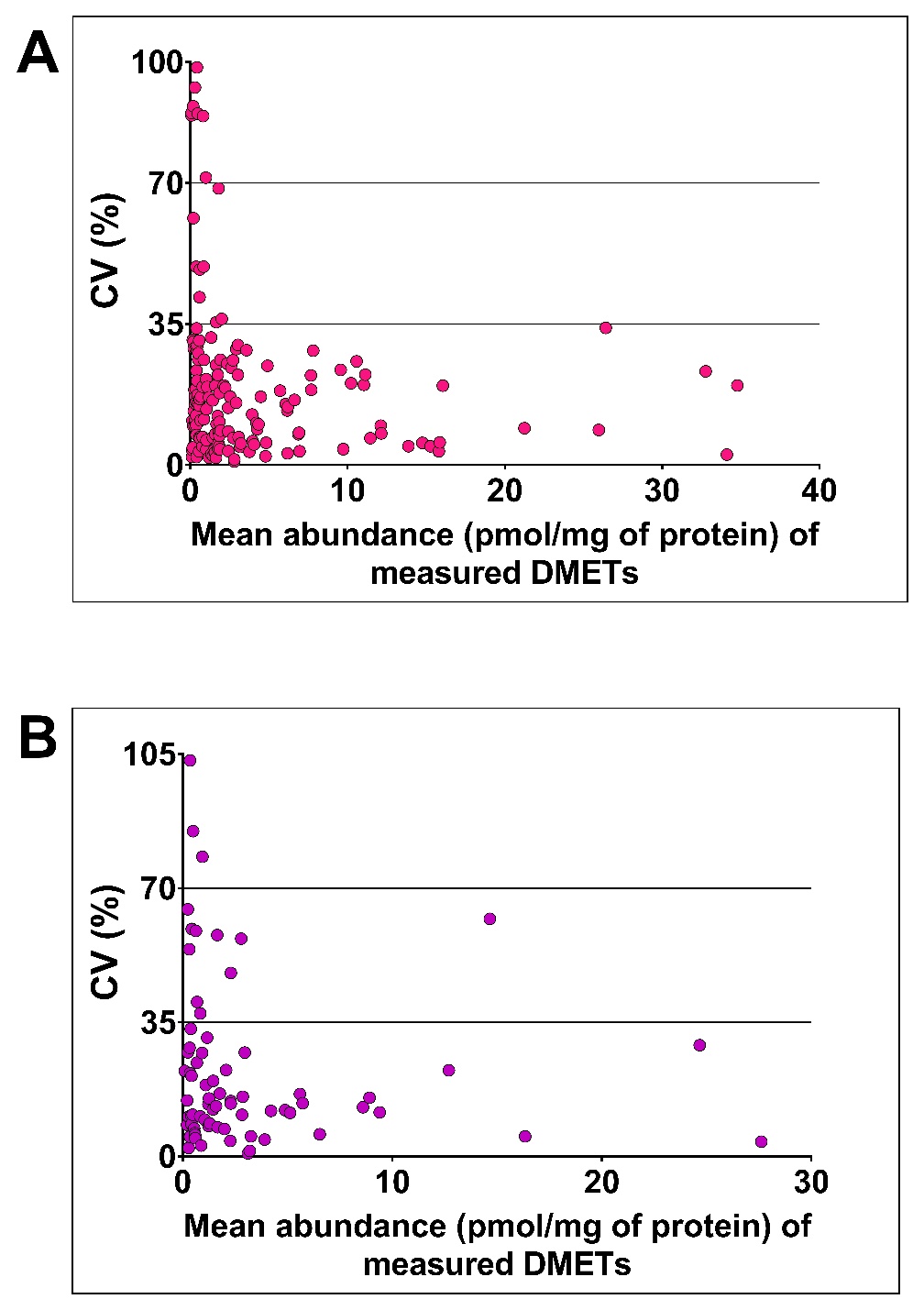


***Figure S5.*** Relative change in expression of DMEs (CYPs, UGTs, SULTs and other enzymes) in healthy ileum samples (n=5), inflamed CD ileum (n=6) and histologically normal CD ileum (n=2). Change in expression is shown for (A) inflamed relative to healthy, (B) histologically normal relative to healthy and (C) inflamed relative to histologically normal. Only targets with fold change ≥2 are reported.


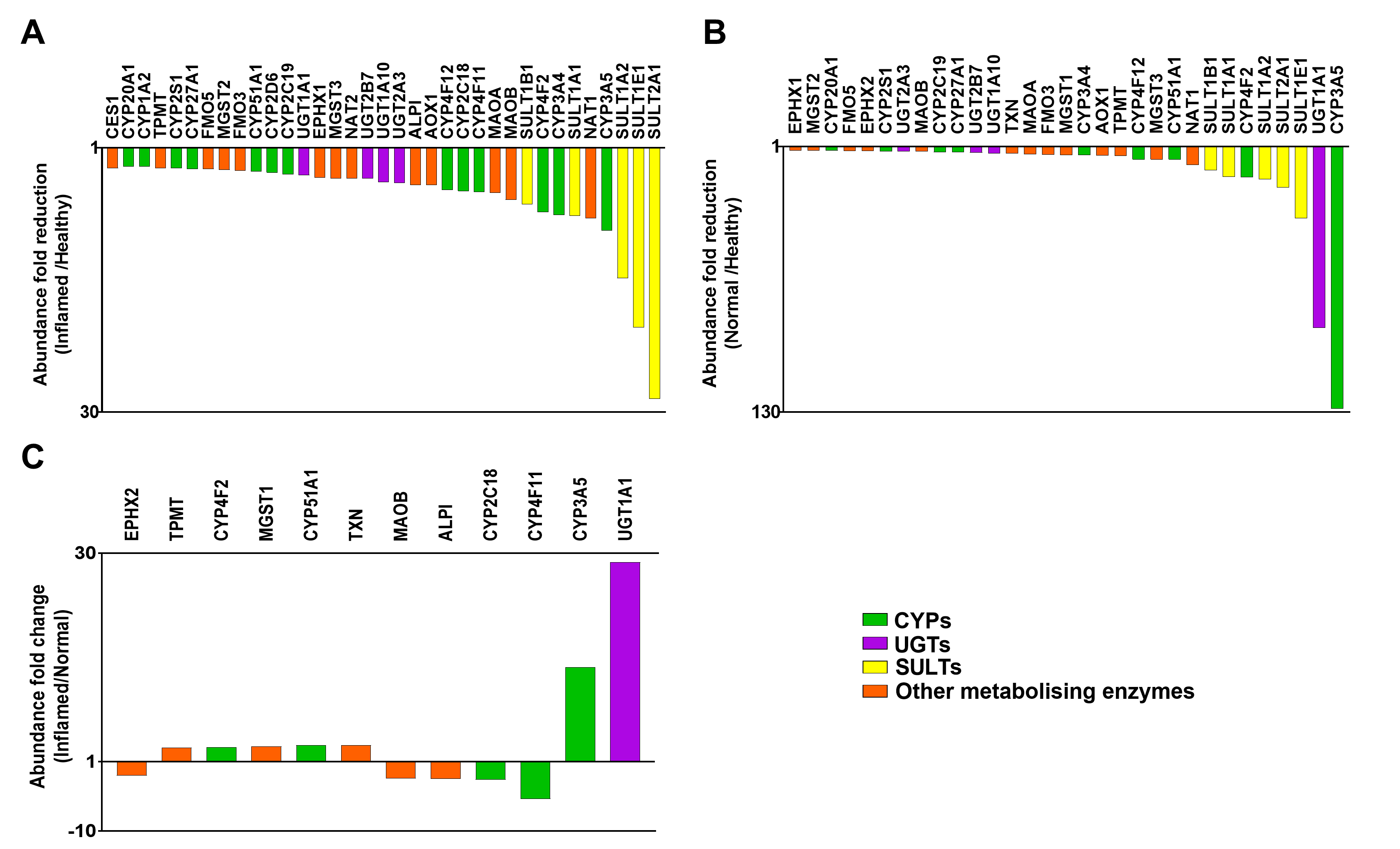


***Figure S6.*** Relative change in expression of drug transporters in healthy ileum samples (n=5), inflamed CD ileum (n=6) and histologically normal CD ileum (n=2). Change in expression is shown for (A) inflamed relative to healthy, (B) histologically normal relative to healthy and (C) inflamed relative to histologically normal. Only targets with fold change ≥2 are reported.


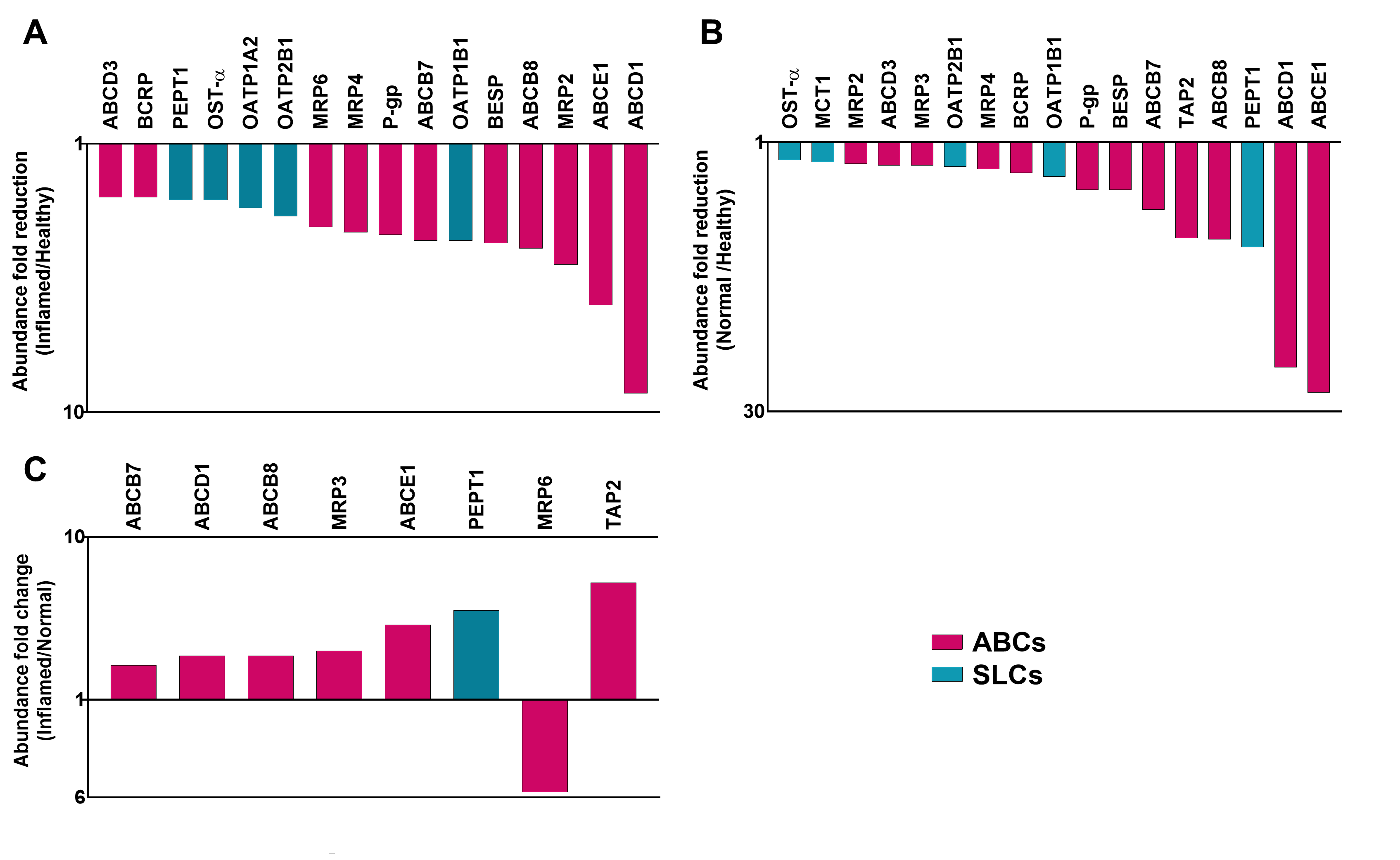


***Figure S7.*** Principal components analysis (PCA) for similarity data based on (**A**) percentage identical peptides (PIP) and (**B**) percentage identical proteins (PIPr). Identified peptides and proteins in 13 ileum samples of healthy, inflamed from Crohn’s disease (I-CD) and non-inflamed from Crohn’s disease (HN-CD) ileum tissues.


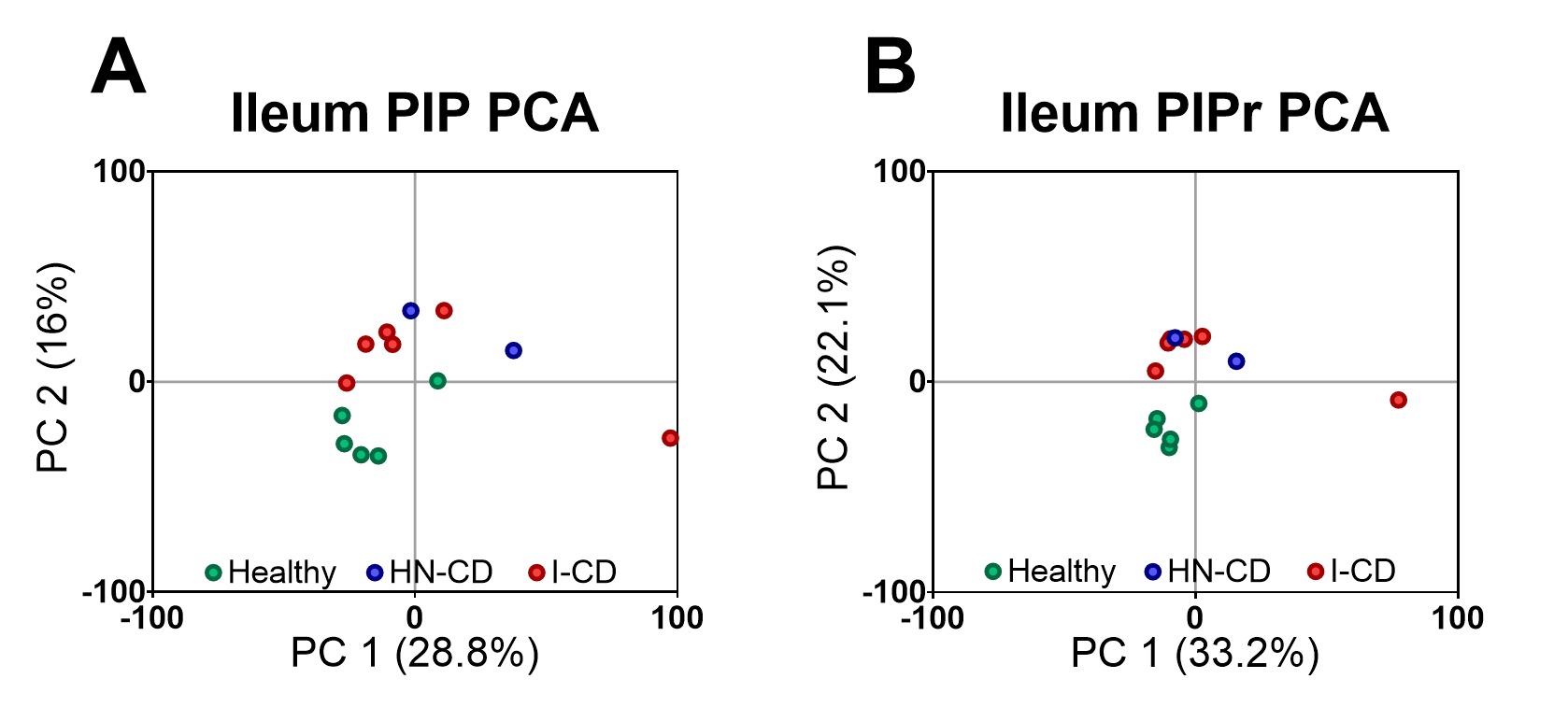


***Figure S8.*** Relative change in expression of DMEs (CYPs, UGTs, SULTs and other enzymes) in healthy colon individual samples (n=5), inflamed CD colon (n=7) and histologically normal CD colon (n=5). Change in expression is shown for (A) inflamed relative to healthy, (B) histologically normal relative to healthy and (C) inflamed relative to histologically normal. Only targets with fold change ≥2 are reported.


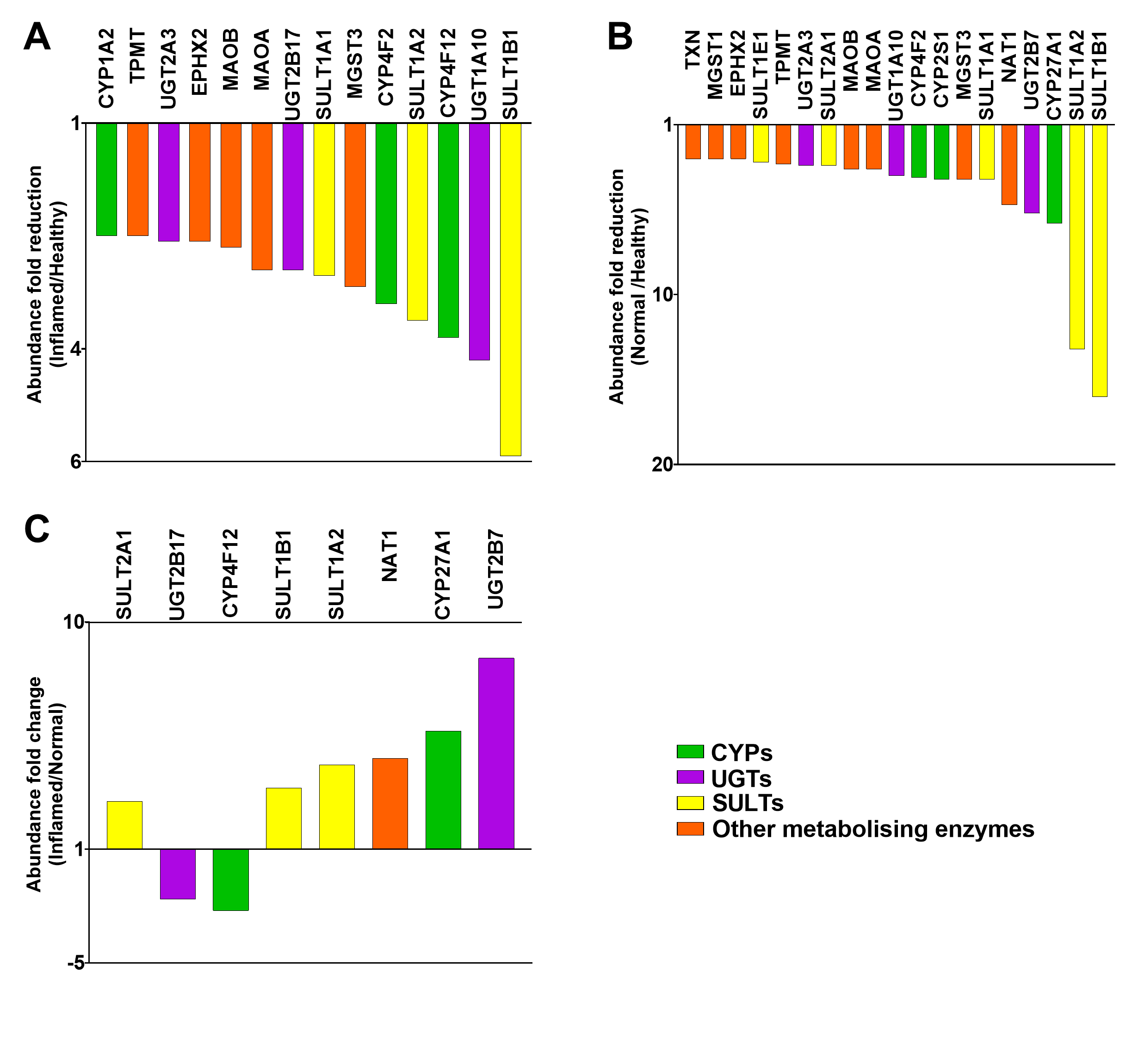


***Figure S9.*** Relative change in expression of drug transporters in healthy colon samples (n=5), inflamed CD colon (n=7) and histologically normal CD colon (n=5). Change in expression is shown for (A) inflamed relative to healthy, (B) histologically normal relative to healthy and (C) inflamed relative to histologically normal. Only targets with fold change ≥2 are reported.


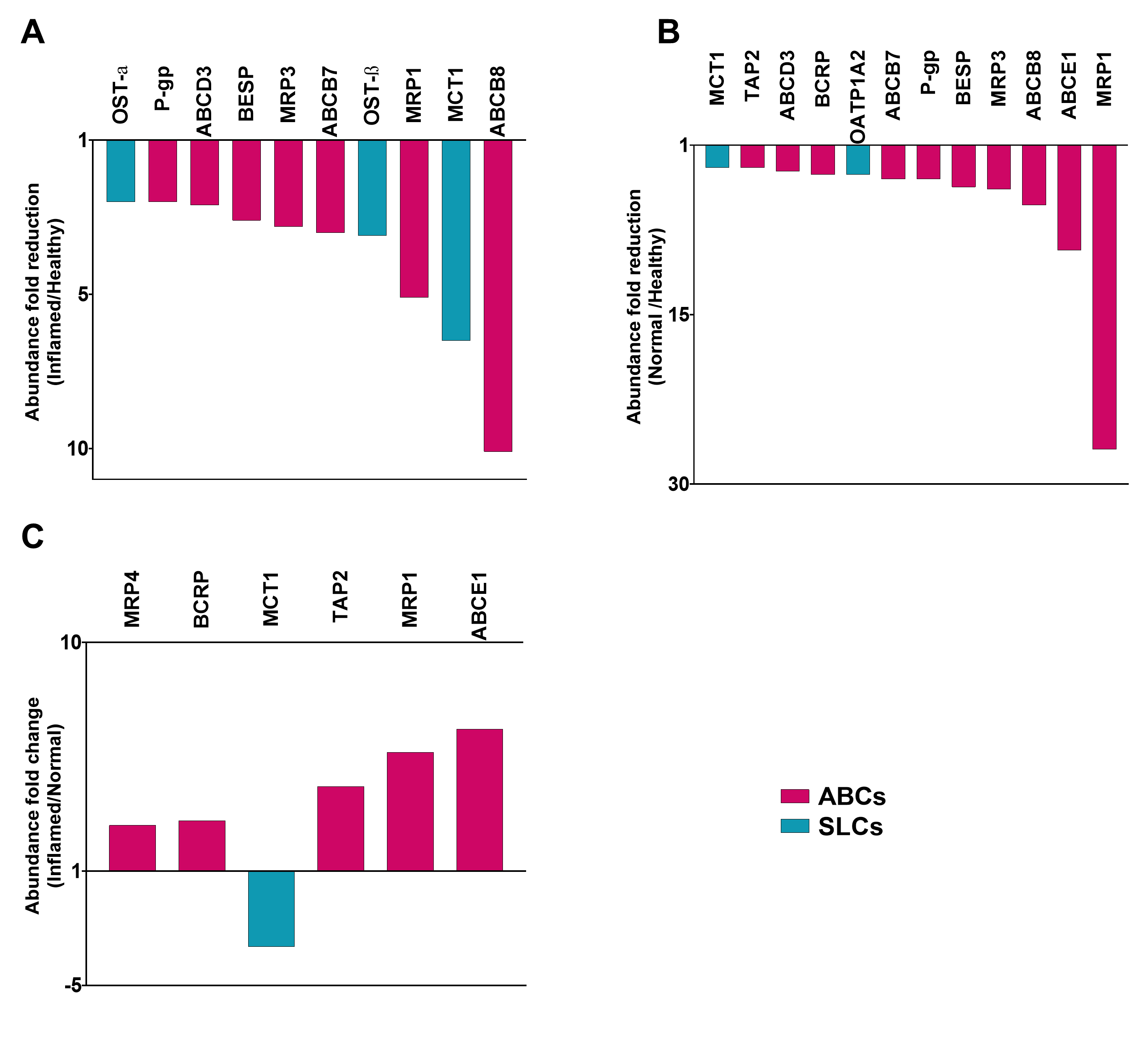

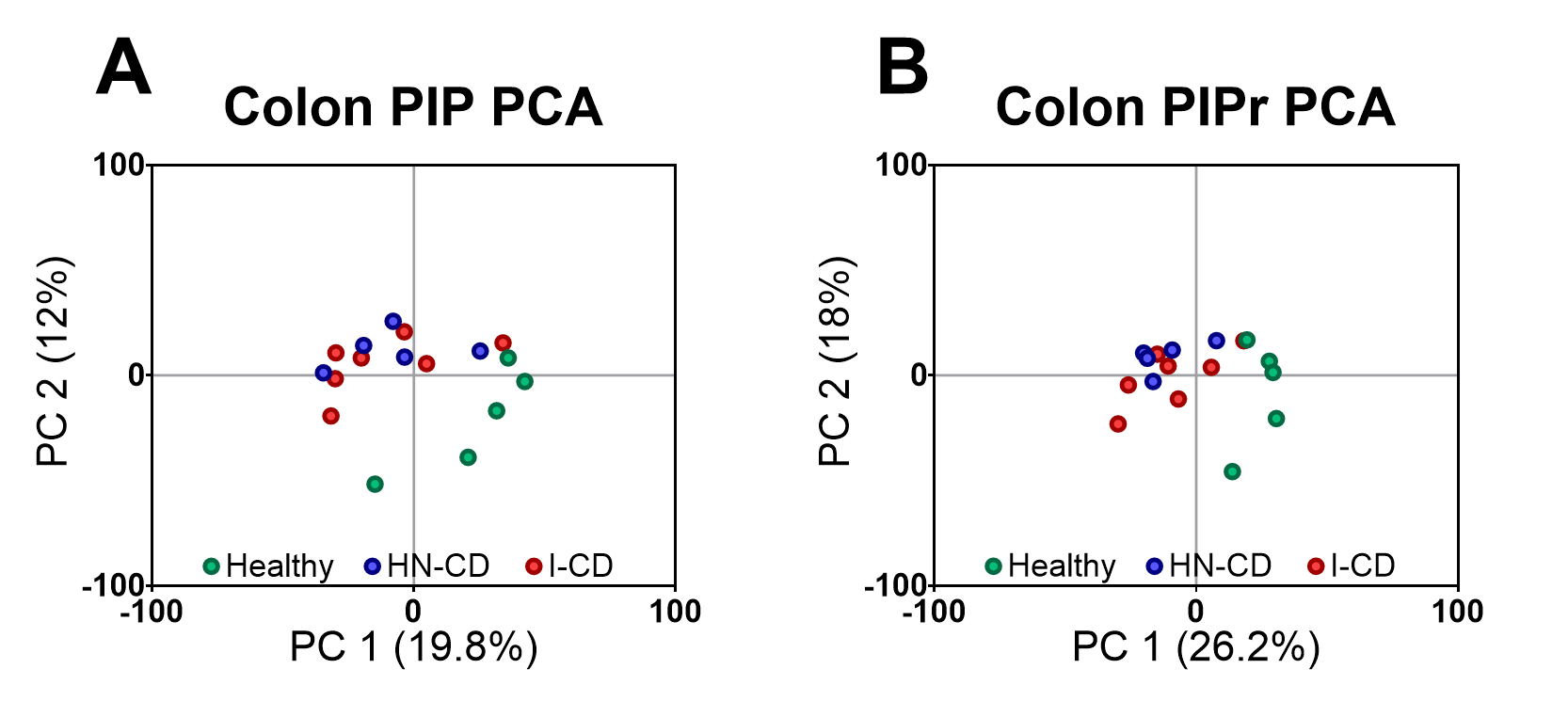


***Figure S10.*** Principal components analysis (PCA) of similarity data based on (**A**) percentage identical peptides (PIP) and (**B**) percentage identical proteins (PIPr). Identified peptides and proteins in 17 colon samples of healthy, inflamed from Crohn’s disease (I-CD) and non-inflamed from Crohn’s disease (HN-CD) colon tissues.

| **M-1** | **Systemic Concentration (nM)** | **Midazolam** | **Budesonide** |
| --- | --- | --- | --- |
|  |  | 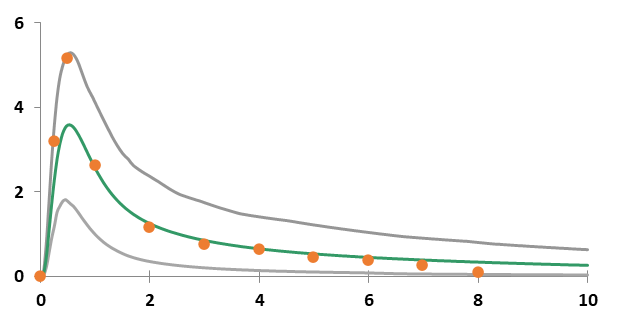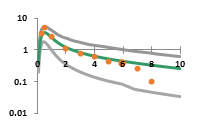 | 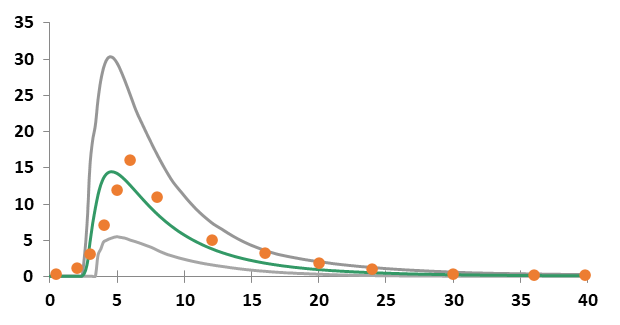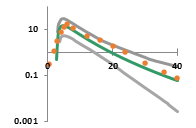 |
| **M-2** |  | 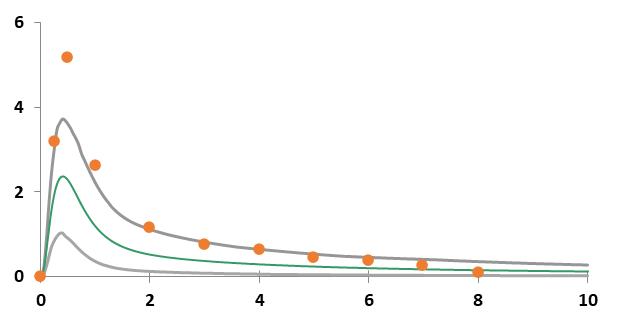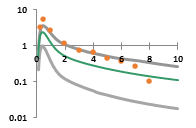 | 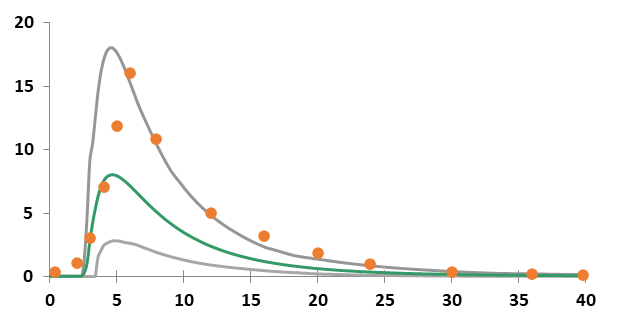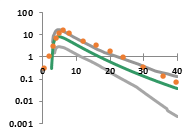 |
| **M-3** |  | 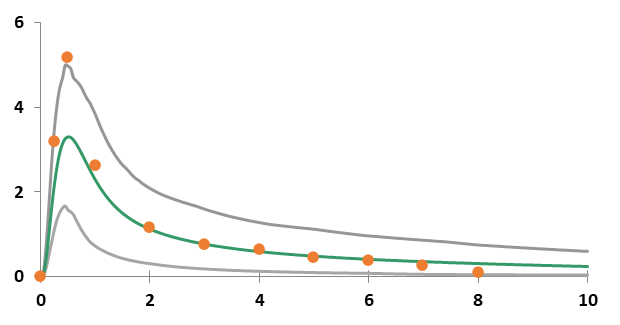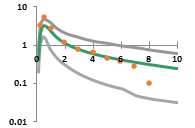 | 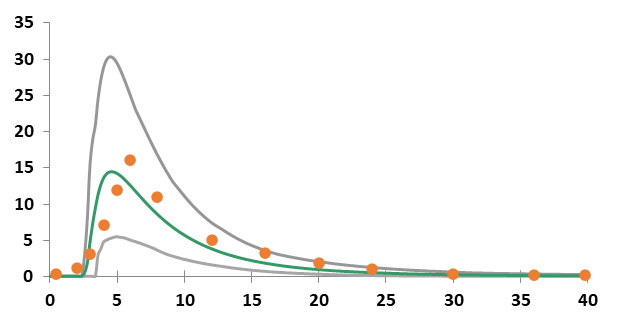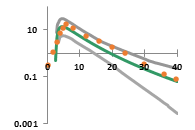 |
| **M-4** |  | 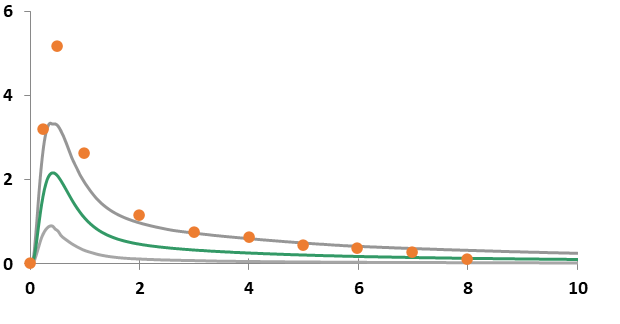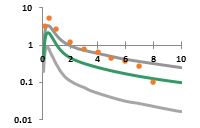 | 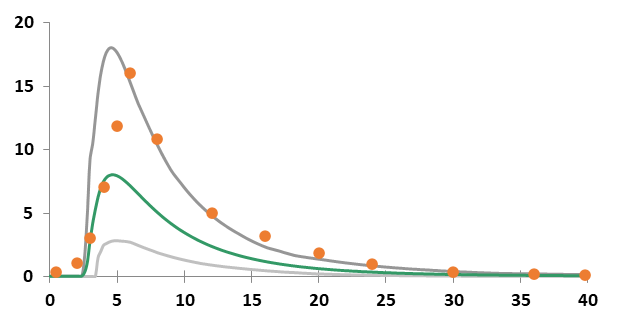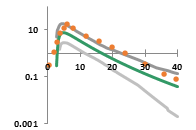 |
|  | **Time (h)** | | |

***Figure S11.*** Simulation of concentration–time profile of midazolam (n=8) following 0.1 mg solution orally in the fasted state^5^ and budesonide (n=6) following 18-mg controlled-release capsule orally in the fed state*)*^4^. Observed data (orange circles) are compared with the 5^th^ and 95^th^ percentile (upper and lower grey lines) of the total virtual population and the mean prediction profile (green central line) generated from physiologically based pharmacokinetic models of active Crohn’s disease population created with metabolising enzymes and transporters abundance values generated in this study and other systems changes^9^ (M-1; intestine DMET abundance data from I-CD tissues and normal albumin level, M-2; intestine DMET abundance data from I-CD tissues and reduced albumin level, M-3; intestine DMET abundance data from HN-CD tissues and normal albumin level and M-4; intestine DMET abundance data from HN-CD tissues and reduced albumin level.

## **Supplementary References**

1. Couto N, Al-Majdoub ZM, Gibson S, et al. Quantitative Proteomics of Clinically Relevant Drug-Metabolizing Enzymes and Drug Transporters and Their Intercorrelations in the Human Small Intestine. *Drug Metabolism and Disposition*. 2020;48(4):245-254. doi:10.1124/dmd.119.089656

2. El-Khateeb E, Al-Majdoub ZM, Rostami-Hodjegan A, Barber J, Achour B. Proteomic Quantification of Changes in Abundance of Drug-Metabolizing Enzymes and Drug Transporters in Human Liver Cirrhosis: Different Methods, Similar Outcomes. *Drug Metabolism and Disposition*. 2021;49(8):610-618. doi:10.1124/dmd.121.000484

3. Chevreux G, Tilly N, Bihoreau N. Quantification of proteins by data independent acquisition: Performance assessment of the Hi3 methodology. *Anal Biochem*. 2018;549:184-187. doi:10.1016/j.ab.2018.03.019

4. Edsbäcker S, Bengtsson B, Larsson P, et al. A pharmacoscintigraphic evaluation of oral budesonide given as controlled-release (Entocort) capsules. *Aliment Pharmacol Ther*. 2003;17(4):525-536. doi:10.1046/j.1365-2036.2003.01426.x

5. Wilson A, Tirona RG, Kim RB. CYP3A4 Activity is Markedly Lower in Patients with Crohnʼs Disease. *Inflamm Bowel Dis*. 2017;23(5):804-813. doi:10.1097/MIB.0000000000001062

6. Sunkara G, Yeh C, Saylan ML, Kawashita H, Koseki N. Assessment of Ethnic Differences in the Pharmacokinetics and Pharmacodynamics of Valsartan. *J Bioequivalence Bioavailab*. 2010;2(6):120-124. doi:10.4172/jbb.1000043

7. Thorsson L, Edsbäcker S, Conradson TB. Lung deposition of budesonide from Turbuhaler® is twice that from a pressurized metered-dose inhaler P-MDI. *European Respiratory Journal*. 1994;7(10):1839-1844. doi:10.1183/09031936.94.07101839

8. Hohmann N, Kocheise F, Carls A, Burhenne J, Haefeli WE, Mikus G. Midazolam microdose to determine systemic and pre-systemic metabolic CYP3A activity in humans. *Br J Clin Pharmacol*. 2015;79(2):278-285. doi:10.1111/bcp.12502

9. Alrubia S, Mao J, Chen Y, Barber J, Rostami-Hodjegan A. Altered Bioavailability and Pharmacokinetics in Crohn’s Disease: Capturing Systems Parameters for PBPK to Assist with Predicting the Fate of Orally Administered Drugs. *Clin Pharmacokinet*. 2022;61(10):1365-1392. doi:10.1007/s40262-022-01169-4
